# Plasma lipids and risk of aortic valve stenosis: a Mendelian randomization study

**DOI:** 10.1093/eurheartj/ehaa070

**Published:** 2020-02-20

**Authors:** Milad Nazarzadeh, Ana-Catarina Pinho-Gomes, Zeinab Bidel, Abbas Dehghan, Dexter Canoy, Abdelaali Hassaine, Jose Roberto Ayala Solares, Gholamreza Salimi-Khorshidi, George Davey Smith, Catherine M Otto, Kazem Rahimi

**Affiliations:** The George Institute for Global Health, University of Oxford, 1st Floor, Hayes House, 75 George Street, Oxford OX1 2BQ, UK; Deep Medicine, Oxford Martin School, University of Oxford, Oxford, UK; The Collaboration Center of Meta-Analysis Research, School of Health, Torbat Heydariyeh University of Medical Sciences, Torbat Heydariyeh, Iran; The George Institute for Global Health, University of Oxford, 1st Floor, Hayes House, 75 George Street, Oxford OX1 2BQ, UK; Deep Medicine, Oxford Martin School, University of Oxford, Oxford, UK; The George Institute for Global Health, University of Oxford, 1st Floor, Hayes House, 75 George Street, Oxford OX1 2BQ, UK; Deep Medicine, Oxford Martin School, University of Oxford, Oxford, UK; The Collaboration Center of Meta-Analysis Research, School of Health, Torbat Heydariyeh University of Medical Sciences, Torbat Heydariyeh, Iran; Department of Biostatistics and Epidemiology, School of Public Health, Imperial College London, London, UK; The George Institute for Global Health, University of Oxford, 1st Floor, Hayes House, 75 George Street, Oxford OX1 2BQ, UK; Deep Medicine, Oxford Martin School, University of Oxford, Oxford, UK; NIHR Oxford Biomedical Research Centre, Oxford University Hospitals NHS Foundation Trust, Oxford, UK; Faculty of Medicine, University of New South Wales, Sydney, Australia; The George Institute for Global Health, University of Oxford, 1st Floor, Hayes House, 75 George Street, Oxford OX1 2BQ, UK; Deep Medicine, Oxford Martin School, University of Oxford, Oxford, UK; The George Institute for Global Health, University of Oxford, 1st Floor, Hayes House, 75 George Street, Oxford OX1 2BQ, UK; Deep Medicine, Oxford Martin School, University of Oxford, Oxford, UK; The George Institute for Global Health, University of Oxford, 1st Floor, Hayes House, 75 George Street, Oxford OX1 2BQ, UK; Deep Medicine, Oxford Martin School, University of Oxford, Oxford, UK; MRC Integrative Epidemiology Unit, University of Bristol, Bristol, UK; University of Washington, Seattle, WA, USA; The George Institute for Global Health, University of Oxford, 1st Floor, Hayes House, 75 George Street, Oxford OX1 2BQ, UK; Deep Medicine, Oxford Martin School, University of Oxford, Oxford, UK; NIHR Oxford Biomedical Research Centre, Oxford University Hospitals NHS Foundation Trust, Oxford, UK

**Keywords:** Blood cholesterol, Lipid profile, Heart valve diseases, Mendelian randomization analysis

## Abstract

**Aims:**

Aortic valve stenosis is commonly considered a degenerative disorder with no recommended preventive intervention, with only valve replacement surgery or catheter intervention as treatment options. We sought to assess the causal association between exposure to lipid levels and risk of aortic stenosis.

**Methods and results:**

Causality of association was assessed using two-sample Mendelian randomization framework through different statistical methods. We retrieved summary estimations of 157 genetic variants that have been shown to be associated with plasma lipid levels in the Global Lipids Genetics Consortium that included 188 577 participants, mostly European ancestry, and genetic association with aortic stenosis as the main outcome from a total of 432 173 participants in the UK Biobank. Secondary negative control outcomes included aortic regurgitation and mitral regurgitation. The odds ratio for developing aortic stenosis per unit increase in lipid parameter was 1.52 [95% confidence interval (CI) 1.22–1.90; per 0.98 mmol/L] for low density lipoprotein (LDL)-cholesterol, 1.03 (95% CI 0.80–1.31; per 0.41 mmol/L) for high density lipoprotein (HDL)-cholesterol, and 1.38 (95% CI 0.92–2.07; per 1 mmol/L) for triglycerides. There was no evidence of a causal association between any of the lipid parameters and aortic or mitral regurgitation.

**Conclusion:**

Lifelong exposure to high LDL-cholesterol increases the risk of symptomatic aortic stenosis, suggesting that LDL-lowering treatment may be effective in its prevention.

**Figure:**
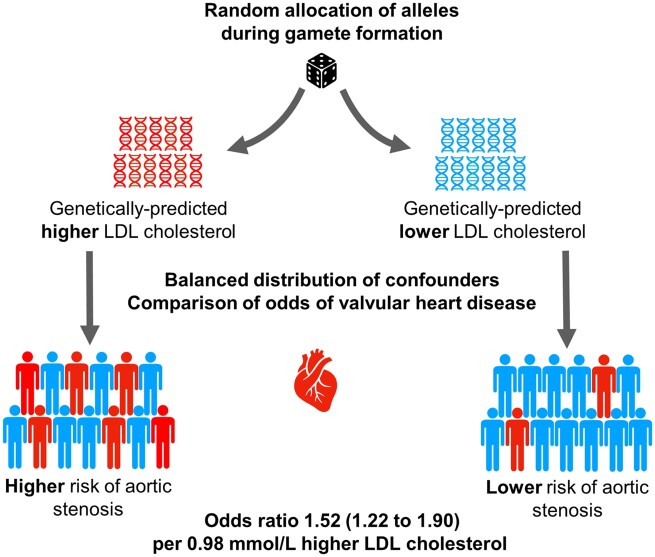

**See page 3921 for the editorial comment on this article (doi: 10.1093/eurheartj/ehaa225)**

## Introduction

A marked shift in the epidemiology of valvular heart disease has been observed in the past century.^1^ Degenerative valve disease, typically manifesting as aortic stenosis or mitral regurgitation, has replaced rheumatic valve disease as the leading cause of valvular heart disease, a trend fuelled by population ageing and increased prevalence of cardiovascular risk factors.^2^ ^,^ ^3^ However, medical treatment for valvular heart disease remains limited and many patients would eventually need valve surgery or catheter-based valve repair or replacement.^4^ Such procedures are associated with significant complications and are costly, with recent estimates suggesting that procedural costs amount to £10 000 and £16 000 for surgical and catheter-based interventions in the UK.^5^

Poor understanding of the underlying mechanisms and risk factors for initiation and progression of valvular heart disease has hindered the development of effective medical treatment for primary and secondary prevention. Considering the shared aetiological pathways between different types of cardiovascular disease,^6–8^ several risk factors have been investigated, but findings for dyslipidaemia have been inconsistent. Whilst observational studies have suggested a potential association between dyslipidaemia and risk of aortic stenosis,^9^ randomized controlled trials (RCTs) have not demonstrated any effect of statin therapy on progression of aortic stenosis.^10–12^ However, RCTs have been based on mostly small sample sizes, relatively short follow-up, and inclusion of patients with established disease.

With this limited evidence from observational and interventional studies, Mendelian randomization (MR) offers an opportunity to efficiently and reliably investigate the potential causal association between dyslipidaemia and valvular heart disease. Mendelian randomization uses instrumental variable analysis to mimic the randomization process that underpins causal inference in RCTs. It is an approach that takes advantage of the naturally occurring random allocation of alleles inherited by offspring from their parents during the formation of the zygote (Supplementary material online, *Figure S1*). This process is similar to the random allocation of treatment in RCTs and could therefore overcome the problems of reverse causation and confounding inherent in observational studies.^13^ We aimed to use MR techniques to test the hypothesis that elevated plasma lipids are causally related to the risk of incident aortic stenosis.

## Methods

### Data for exposure

Our main exposure was genetically determined plasma lipids as instrumental variable. This was estimated from genetic variants that were associated with levels of low density lipoprotein (LDL)-cholesterol, high density lipoprotein (HDL)-cholesterol, triglycerides, and total cholesterol at genome-wide significance level. We retrieved summary estimations of 157 genetic variants that have been shown: (i) to be associated with plasma lipid levels in the Global Lipids Genetics Consortium (GLGC) genome-wide association study (*P *<* *5 × 10^−8^) that included 188 577 participants, mostly European ancestry and (ii) were independently associated with plasma lipid levels (linkage disequilibrium threshold of *r* ^2^ < 0.01 and located 1 Mb apart from each other) (Supplementary material online, *Datasets S1–S4*).^14^ A detailed description of the statistical methods and quality control is provided in a previous publication by the GLGC.^14^ Briefly, as in most studies included in the GLGC, plasma lipid concentrations had been measured after at least 8 h fasting, and the estimations were adjusted for age, age squared, sex, and population stratification. Participants with known lipid-lowering medication use have been excluded from study.^14^ Selected genetic variants together explained 10–14% of the total trait variance.^14^ Additive genetic models using linear regression on the inverse normal transformed traits were fitted for individual variant association estimates, and a weighted meta-analysis using Stouffer method was conducted for combined estimates.^14^ The effect sizes were calculated with respect to the minor allele per 1 SD increase in plasma lipid levels (1 SD is equal to 0.98 mmol/L for LDL-cholesterol, 0.41 mmol/L for HDL-cholesterol, 1 mmol/L for triglycerides, and 1.10 mmol/L for total cholesterol).^14^

### Data for outcome

We used the UK Biobank data, a large prospective cohort study including 502 602 participants aged 40–69 years and recruited between 2006 and 2010 from 22 assessment centres across the UK. Details of the study design have been published elsewhere.^15^ ^,^ ^16^ UK Biobank genotype data were imputed with IMPUTE4 using the Haplotype Reference Consortium and the UK10K + 1000 Genomes panel^17^ to identify ∼96 million variants for 487 381 participants. We excluded 55 208 individuals who were outliers based on heterozygosity, had a variant call rate <98%, or were not recorded as ‘white British’. The remaining participants (*n* = 432 173) were included in the estimation of genetic variants-outcome association in this study. The protocol of the present study was approved by UK Biobank (#22207).

Aortic stenosis was the primary outcome, with aortic regurgitation and mitral regurgitation as the negative control secondary outcomes (Supplementary material online, *Text S1*). We calculated corresponding summary statistics for the outcomes using logistic regression model adjusted for age, sex, assessment centre, genetic batch, the first 10 genetic principal component (for addressing population stratification), and up to third-degree relatedness based on kinship coefficients (>0.044).

### Statistical analysis

#### Two-sample Mendelian randomization to assess total causal effect

We harmonized summary data based on a previously described method.^18^ Then, we used four different methods of two-sample MR [inverse-variance weighted (random-effects model), weighted median, MR-Egger, and MR-PRESSO] in order to address between variants heterogeneity and pleiotropy effect. The inverse-variance weighted method assumes that either all the instruments are valid or any horizontal pleiotropy is balanced.^19^ We provided an estimation using the weighted median method, which is consistent if at least 50% of the weight comes from valid instrumental variables.^20^ The MR-Egger regression method was used as the main estimation to account for potential pleiotropy.^21^ In addition, the MR pleiotropy residual sum and outlier (MR-PRESSO) method was used to test, and correct, if needed, for possible horizontal pleiotropic outliers in the analysis.^22^

We considered the association as causal when at least three methods provided consistent results. This approach reduces the risk of false-positive interpretation, and demonstration of consistent findings across the various models is likely to strengthen the case for a causal association. We used a predefined approach to select the best statistical estimation from these four methods (see Supplementary material online, *Figure S2* for details). A leave-one-out sensitivity analysis was conducted by removing a single variant from the analysis in turn. The fluctuation of the estimates in response to excluding each variant reflects the possibility of outlier variant in the causal estimation. We examined the heterogeneity of the estimates using a scatter plot and applying the Cochran’s *Q*-test.^23^ We also assessed the probable directional pleiotropy using a funnel plot similar to that being used to assess for publication bias in meta-analysis.^23^

The minimum detectable odds ratio (OR) was calculated using the method reported by Brion *et al*.^24^, and implemented in a web-based application (Supplementary material online, *Table S1*). In addition to using negative control outcomes, we tested the validity of the instrumental variable by examining the causal association between plasma lipids and coronary heart disease as a positive outcome for LDL-cholesterol, total cholesterol, and triglycerides and a negative outcome for HDL-cholesterol.^25^ For this control analysis, we used two-sample MR using an analytical platform.^26^ We used the same genetic variants for plasma lipids, but the variants-outcome association was extracted from a large genome-wide association study meta-analysis including 22 233 individuals with coronary heart disease and 64 762 controls of European population.^27^ To address the possible mediating effect of myocardial infarction and heart failure on the association between lipid profile and aortic stenosis, we performed sensitivity analysis that excluded individuals with myocardial infarction and/or heart failure. In addition, to assess the robustness of the findings, we restricted cases to those with aortic stenosis and aortic valve replacement surgery. All the statistical analyses were performed using R software (‘MendelianRandomization’^28^ and ‘TwoSampleMR’^26^ packages).

#### Multivariable Mendelian randomization to assess the direct causal effect

We used multivariable MR through inverse-variance weighted method to estimate the direct causal effect of lipid profile on the outcomes. We excluded total cholesterol from this analysis because of observable overlap between total and LDL-cholesterol. In cases where the exposures of interest are correlated, such as total and LDL-cholesterol, the multivariable MR is useful to estimate direct causal effect of each lipid profile component, independently of any other lipid profile variables.^29^ ^,^ ^30^ Given that a causal link between elevated lipoprotein-a [LP(a)] and aortic stenosis has been reported,^31^ we repeated the multivariable MR additionally adjusted for LP(a) to further check the possible effect of LP(a) on the associations. The biochemistry and genetic data for LP(a) have been obtained from the UK Biobank resource.

## Results

### Main findings

**Figure 1:**
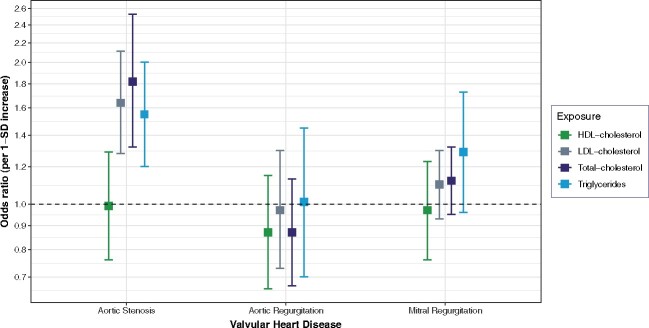
Comparison of the total causal estimations considered heterogeneity and pleiotropic effect between plasma lipids and valvular heart disease risk using two-sample Mendelian randomization. SD, standard deviation; 1 SD is equal to 0.98 mmol/L for low density lipoprotein-cholesterol, 0.41 mmol/L for high density lipoprotein-cholesterol, 1 mmol/L for triglycerides, and 1.10 mmol/L for total cholesterol.

The characteristics of the populations included in the GLGC and UK Biobank are shown in *Table 1*. In the UK Biobank, we identified 1961 participants with aortic stenosis, 736 with aortic regurgitation, and 2213 with mitral regurgitation. *Table 2* shows the results of MR for aortic stenosis. There was clear evidence of a causal effect of LDL-cholesterol, total cholesterol, and triglycerides on aortic stenosis (*P* < 0.05 in the three MR methods) (*Table 2* and Supplementary material online, *Figure S3*). Considering the best causal estimation, the OR was 1.64 [95% confidence interval (CI) 1.28–2.11] per 0.98 mmol/L increase in LDL-cholesterol, 1.82 (95% CI 1.32–2.53) per 1.10 mmol/L increase in total cholesterol, and 1.55 (95% CI 1.20–2.00) per 1 mmol/L increase in triglycerides. The findings were also concordant on the lack of association between HDL-cholesterol and aortic stenosis. There was no evidence in favour of an association between plasma lipid parameters and aortic or mitral regurgitation, other than for a weak association between triglycerides and mitral regurgitation (*Tables 3 and 4*). However, the latter finding was only supported by one of the methods whilst all other methods consistently showed null associations between all lipid parameters and aortic and mitral regurgitation (Supplementary material online, *Figure S3*). *Figure 1* compares the risk estimates from the MR analyses separately for each outcome. There was no evidence of directional pleiotropy except for total cholesterol (beta = −0.009; *P* = 0.04 in MR-Egger intercept) (Table 2). The funnel plots show an absence of directional pleiotropy, with a symmetrical distribution of variants effects (Supplementary material online, *Figures S4–S11*). However, there was significant heterogeneity for all lipid parameters. The control analysis with coronary heart disease as the outcome showed positive and significant association with each of lipid parameters other than for HDL-cholesterol, confirming that the selected genetic variants were valid instruments (Supplementary material online, *Figure S12*).

**Table 1:** Characteristics of Global Lipids Genetics Consortium and UK Biobank datasets

Exposures	Consortium	No. SNPs	Sample size	Population
HDL-cholesterol	GLGC	71	92 860	90% European
LDL-cholesterol		57	83 198	
Total cholesterol		73	92 260	
Triglycerides		40	91 598	

**Main outcomes**	**Dataset**		**No. cases/sample size**	**Population**

Aortic stenosis	UK Biobank		1961/432 173	100% European
Aortic regurgitation			736/432 173	
Mitral regurgitation			2213/432 173	
Outcomes for sensitivity analysis				
Myocardial infarction			15 391/432 173	
Heart failure			5161/432 173	
Aortic valve replacement			1233/432 173	
Demographic variables				
Age (years), mean (SD)			56.8 (8.0)	
Male gender, *n* (%)			198 623 (45.9)	

GLGC, Global Lipids Genetics Consortium; HDL, high density lipoprotein; LDL, low density lipoprotein; SD, standard deviation; SNP, single nucleotide polymorphism.

**Table 2:** Two-sample Mendelian randomization estimations showing the effect of plasma lipids on aortic stenosis

Methods	Exposure	Odds ratio^a^	95% CI		*P*-value	*P_h_*	*Q*-statistics
Inverse-variance weighted	HDL-cholesterol	0.86	0.69	1.06	0.17	<0.001	117.1
MR-Egger		0.98	0.70	1.37	0.91		
**Weighted median**		**0.99**	**0.76**	**1.29**	**0.93**		
MR-PRESSO^b^		NA	NA	NA	NA		
MR-Egger intercept^c^		−0.009	−0.026	0.008	0.31		

Inverse-variance weighted	LDL-cholesterol	1.58	1.30	1.91	<0.001	0.01	81.6
MR-Egger		1.63	1.19	2.24	<0.001		
**Weighted median**		**1.64**	**1.28**	**2.11**	**<0.001**		
MR-PRESSO		1.59	1.34	1.90	<0.001		
MR-Egger intercept^c^		−0.002	−0.022	0.017	0.80		

Inverse-variance weighted	Total cholesterol	1.60	1.33	1.92	<0.001	0.04	93.7
**MR-Egger**		**1.82**	**1.32**	**2.53**	**<0.001**		
Weighted median		1.73	1.33	2.25	<0.001		
MR-PRESSO^b^		NA	NA	NA	NA		
MR-Egger intercept^c^		−0.009	−0.026	0.009	0.04		

Inverse-variance weighted	Triglycerides	1.52	1.12	2.03	0.006	<0.001	77.5
MR-Egger		1.49	0.95	2.33	0.08		
Weighted median		1.39	1.00	1.92	0.05		
**MR-PRESSO**		**1.55**	**1.20**	**2.00**	**0.002**		
MR-Egger intercept^c^		0.001	−0.024	0.026	0.91		

The best causal estimation highlighted in bold .

CI, confidence interval; HDL, high density lipoprotein; LDL, low density lipoprotein; NA, not applicable; *P_h_*, *P*-value for heterogeneity.

a Odds ratio per 1 SD increase.

b No significant outliers.

c Regression coefficient (95% CI).

**Table 3:** Two-sample Mendelian randomization estimations showing the effect of plasma lipids on aortic regurgitation

Methods	Exposure	Odds ratio^a^	95% CI		*P*-value	*P_h_*	*Q*-statistics
**Inverse-variance weighted**	HDL-cholesterol	**0.87**	**0.66**	**1.15**	**0.35**	0.40	72.1
MR-Egger		0.82	0.53	1.25	0.35		
Weighted median		0.73	0.47	1.13	0.15		
MR-PRESSO^b^		NA	NA	NA	NA		
MR-Egger intercept^c^		0.005	−0.017	0.027	0.65		

**Inverse-variance weighted**	LDL-cholesterol	**0.97**	**0.73**	**1.30**	**0.88**	0.09	70.2
MR-Egger		0.94	0.59	1.51	0.80		
Weighted median		1.10	0.73	1.66	0.63		
MR-PRESSO^b^		NA	NA	NA	NA		
MR-Egger intercept^c^		0.003	−0.027	0.33	0.83		

**Inverse-variance weighted**	Total cholesterol	**0.87**	**0.67**	**1.13**	**0.32**	0.51	70.9
MR-Egger		1.06	0.67	1.69	0.80		
Weighted median		1.11	0.74	1.69	0.61		
MR-PRESSO^b^		NA	NA	NA	NA		
MR-Egger intercept^c^		−0.013	−0.038	0.013	0.33		

**Inverse-variance weighted**	Triglycerides	**1.01**	**0.70**	**1.45**	**0.94**	0.19	46.4
MR-Egger		1.21	0.70	2.09	0.50		
Weighted median		1.26	0.77	2.06	0.36		
MR-PRESSO^b^		NA	NA	NA	NA		
MR-Egger intercept^c^		−0.013	−0.044	0.018	0.39		

The best causal estimation highlighted in bold.

CI, confidence interval; HDL, high density lipoprotein; LDL, low density lipoprotein; NA, not applicable; *P_h_*, *P*-value for heterogeneity.

a Odds ratio per 1 SD increase.

b No significant outliers.

c Regression coefficient (95% CI).

**Table 4:** Two-sample Mendelian randomization estimations showing the effect of plasma lipids on mitral regurgitation

Methods	Exposure	Odds ratio^a^	95% CI		*P*-value	*P_h_*	*Q*-statistics
Inverse-variance weighted	HDL-cholesterol	0.84	0.70	1.02	0.08	0.0009	100.4
MR-Egger		0.96	0.72	1.29	0.80		
**Weighted median**		**0.97**	**0.76**	**1.23**	**0.79**		
MR-PRESSO^b^		NA	NA	NA	NA		
MR-Egger intercept^c^		−0.009	−0.024	0.006	0.25		

**Inverse-variance weighted**	LDL-cholesterol	**1.10**	**0.93**	**1.30**	**0.23**	0.12	68.5
MR-Egger		1.07	0.81	1.40	0.65		
Weighted median		1.08	0.85	1.37	0.52		
MR-PRESSO^b^		NA	NA	NA	NA		
MR-Egger intercept^c^		0.003	−0.014	0.020	0.73		

**Inverse-variance weighted**	Total cholesterol	**1.12**	**0.95**	**1.32**	**0.14**	0.14	84.7
MR-Egger		1.19	0.88	1.60	0.24		
Weighted median		1.08	0.85	1.39	0.50		
MR-PRESSO^b^		NA	NA	NA	NA		
MR-Egger intercept^c^		−0.004	−0.020	0.012	0.65		

Inverse-variance weighted	Triglycerides	1.31	1.04	1.65	0.02	0.05	54.0
MR-Egger		1.30	0.92	1.85	0.13		
**Weighted median**		**1.29**	**0.96**	**1.73**	**0.09**		
MR-PRESSO^b^		NA	NA	NA	NA		
MR-Egger intercept^c^		0.000	−0.019	0.020	0.97		

The best causal estimation highlighted in bold.

CI, confidence interval; HDL, high density lipoprotein; LDL, low density lipoprotein; NA, not applicable; *P_h_*, *P*-value for heterogeneity.

a Odds ratio per 1 SD increase.

b No significant outliers.

c Regression coefficient (95% CI).

### Sensitivity analysis

To assess the potential mediating effect of a presentation with myocardial infarction or heart failure on detection of valve disease, we repeated the analysis excluding all the participants with documented myocardial infarction and/or heart failure. The findings were broadly similar to the overall analysis, other than for a reduction in pleiotropy in total cholesterol analysis after excluded participants with myocardial infarction (MR-Egger intercept = −0.005; *P* = 0.59) (Supplementary material online, *Tables S2–S4 and Figure S13*). There were also no substantial differences in the results after excluding participants with heart failure (Supplementary material online, *Figure S14*). Sensitivity analysis by restricting the outcome only to include those with valve replacement therapy was consistent with the main results (Supplementary material online, *Figure S15*). In the leave-one-out analysis, we found that no single genetic variant was strongly driving the overall effect of plasma lipids on aortic stenosis (Supplementary material online, *Figures S16–S19*).

**Figure 2:**
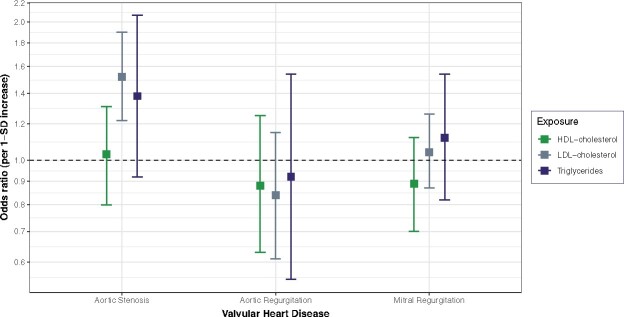
Comparison of the direct causal estimations between plasma lipids and valvular heart disease risk using multivariable Mendelian randomization. SD, standard deviation; 1 SD is equal to 0.98 mmol/L for low density lipoprotein-cholesterol, 0.41 mmol/L for high density lipoprotein-cholesterol, 1 mmol/L for triglycerides, and 1.10 mmol/L for total cholesterol. The multivariable Mendelian randomization was adjusted to estimate direct causal effect of each plasma lipids component, independently of any other plasma lipids variables.

In the multivariable MR that adjusted for the effect of each lipid profile component, the strong positive association between LDL-cholesterol and aortic stenosis persisted, whereas the association with triglycerides was attenuated. The multivariable-adjusted ORs were 1.52 (95% CI 1.22–1.90; per 0.98 mmol/L increase) for LDL-cholesterol, 1.38 (95% CI 0.92–2.07; per 1 mmol/L increase) for triglycerides, and 1.03 (95% CI 0.80–1.31; per 0.41 mmol/L increase) for HDL-cholesterol (*Figure 2*). Additional adjustment for LP(a) did not change the results (Supplementary material online, *Figure S20*).

**Take home figure:**
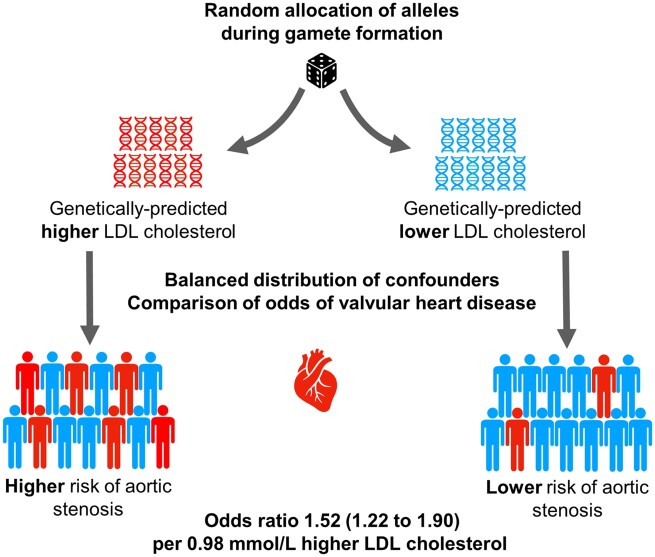
Schematic overview of the Mendelian randomization framework and key findings.

## Discussion

This study showed that each standard deviation increase in LDL-cholesterol, total cholesterol, and triglycerides increases the risk of incident aortic stenosis by 64%, 82%, and 55%, respectively. In contrast, there was no evidence of a causal association between plasma lipids and aortic regurgitation or mitral regurgitation. After adjustment for each lipid profile component through multivariable MR, the result corroborated the association between LDL-cholesterol and risk of aortic stenosis. However, the findings for triglycerides were inconclusive and should be interpreted with caution. This is in part because of the small numbers of independent genetic variants available for triglycerides which could have led to a low statistical power and wide CIs. Nevertheless, the robustness and consistency of our results using different methods, together with the strength of the association, indicate an unconfounded relationship between elevated LDL-cholesterol with the risk of incident aortic stenosis, and suggest that this association is likely to be causal.

This MR study is in keeping with a previous population-based cohort study suggesting that dyslipidaemia was associated with an increased risk of incident aortic stenosis.^9^ However, the observational nature of this earlier report precluded drawing conclusions about causality and the binary categorization of dyslipidaemia limited the study from demonstrating any dose–response relationship. More recently, a one-sample MR study, which included 473 cases of aortic stenosis, demonstrated a causal association between LDL-cholesterol and aortic stenosis, with no evidence of a significant association with triglycerides.^32^ However, the latter may be due to lack of power to detect a small effect size, which is indeed a known limitation when conducting one-sample MR.^33^ Our two-sample analysis, based on data from two non-overlapping datasets, has a higher power than one-sample analysis to detect more modest associations.^34^ In addition, we were able to overcome the issue of weak instrument bias, which may underpin the underestimation of a causal association between triglycerides and aortic stenosis in the aforementioned study.^35^ However, the apparent causal association between raised triglycerides and aortic stenosis was attenuated by adjustment for the effect of other lipid markers. This adjustment substantially reduced the number of variants available as these variants needed to be associated with raised triglycerides but not with cholesterol markers. Therefore, the independent association between raised triglycerides and aortic stenosis remains uncertain. A similar issue is seen when assessing the direct causal effect of triglycerides on coronary heart disease, where the small number of genetic variants precluded precise causal estimation of the association using MR technique.^30^

Our findings are supported by pathophysiological studies which have shown the involvement of an atherosclerotic process of the valve cusps in aortic stenosis, similar to what happens in the arterial tree.^36–38^ It is thus biologically plausible that well-established causal factors in the development of atherosclerosis, particularly in the coronary arteries, maybe also be involved in the pathological process of aortic stenosis.^39^ ^,^ ^40^ Cholesterol, and more specifically, LDL-cholesterol, is a clearly established risk factor of atherosclerotic diseases, whilst the role of triglycerides as an independent risk factor remains controversial.^41–43^ In addition, although experimental studies have suggested that components of HDL particles may have positive effects on aortic stenosis, we did not find an association between genetically determined HDL-cholesterol levels and risk of aortic stenosis.^44^ This is in keeping with the lack of effect of HDL-raising treatments for primary and secondary prevention of coronary artery disease consistently reported by both RCTs^45^ ^,^ ^46^ and MR studies.^47^ Therefore, further evidence is warranted to understand the role of HDL-cholesterol in aortic stenosis pathogenesis and whether increasing HDL-cholesterol level could have a beneficial impact in delaying the disease progression.

To the best of our knowledge, no RCT has yet assessed the effect of lipid modification for primary prevention of aortic stenosis. However, three randomized trials have investigated the effects of statins in patients with mild to moderate aortic stenosis. Although these trials failed to show a clear benefit for LDL-lowering therapy in delaying the progression of aortic stenosis to eventually require aortic valve replacement,^10–12^ ^,^ ^48^ they were mostly limited by a short follow-up duration and insufficient statistical power.^10^ ^,^ ^11^ ^,^ ^48^ Indeed, detecting a relatively small treatment effect on a slowly progressive disease will likely require a substantially large sample size. In addition, our MR analysis reflects the impact of lifelong exposure to higher levels of cholesterol and triglycerides, capturing long-term risks that may not be modifiable by short-term lipid-lowering treatment.^49^ Indeed, it is possible that cholesterol-induced atherosclerosis plays a more important role in initiation than in progression of aortic stenosis, which, for practical reasons, has been the main outcome of earlier RCTs. It is possible that an initial damage to the aortic cups disturbs valve function and flow, setting in motion an irreversible cycle of disturbed flow, abnormal pressure, endothelial damage, and calcification that eventually leads to severe stenosis requiring valve replacement.^50^ Once a certain threshold of valve damage has been crossed, cholesterol-lowering treatment might not be able to halt progression of aortic valve disease. As aortic stenosis has a long, silent clinically asymptomatic phase, it is plausible that treatment initiation after clinical manifestation might be too late to revert the pathologic process that has been triggered by prolonged exposure to raised lipid levels. A large randomized prospective, placebo vs. high-dose statin clinical trial in patients with subclinical aortic sclerosis or mild aortic stenosis would need to be conducted to test whether statin treatment can slow progression to overt aortic stenosis.

Given the established causal link between elevated LP(a) and aortic stenosis,^31^ and evidence showing that statin therapy increases LP(a) levels,^51^ it is also plausible that some of the expected beneficial LDL-lowering effects of statins in previous trials have been counteracted by a concomitant rise in LP(a). However, in our multivariable MR analysis, we adjusted for genetically determined LP(a) yet the risk estimate of LDL-cholesterol on aortic stenosis remained virtually unchanged.

In our study, the estimates used for outcomes are from valvular heart disease cases, which were obtained from linked hospital electronic health records, from which we could not assess disease progression or severity. Disease outcomes may also be affected by a degree of misclassification as we relied on using routinely collected data to identify cases, with no access to echocardiographic data for direct case ascertainment. However, previous studies that relied on electronic health records to identify outcomes have shown that the majority of clinically recorded valve disease codes were based on echocardiographic assessments, and the recorded cases were typically in the moderate to severe spectrum of the disease.^31^ ^,^ ^52^ In addition, restricting cases to those with a valve replacement therapy as a proxy for valve severity yielded similar results. Also, our study assumed that the genetic variants selected as proxy for lipid levels influenced valvular heart disease only through the exposure of interest. Although it is impossible to be certain that the variants used in this study do not have pleiotropic effects, we did not find any evidence in favour of strong pleiotropy. Finally, the current study relied on genetic data conducted in a population mostly of European descent, which, despite the benefit of greater genetic homogeneity, limits the generalizability of the present findings to other ethnicities. It would be interesting to study whether the observed associations hold true in populations with different genetic backgrounds.

In this study, we showed that genetically determined exposure to raised lipid levels, specifically LDL-cholesterol, total cholesterol, and triglycerides, significantly increased the risk of aortic stenosis. There was no evidence that such exposure to raised lipid levels were associated with aortic regurgitation and mitral regurgitation. After adjustment for other lipid components, the finding further confirmed the causal association between LDL-cholesterol and risk of aortic stenosis. In the absence of high-quality evidence from clinical trials, this study provides the most compelling evidence that lipids play a role in the aetiology of aortic stenosis. Considering the substantial ethical and practical implications of conducting large scale RCTs, particularly for primary prevention, this study could guide clinical decision-making regarding lipid-lowering treatment, which may contribute to curb the global epidemic of aortic valve stenosis.

## Supplementary Material

ehaa070_supplementary_data
