# Supplementary material for: Plasma lipids and risk of aortic valve stenosis: a Mendelian randomization study
Source: Eur Heart J. 2020 Feb 20;41(40):3913–20. doi: 10.1093/eurheartj/ehaa070 (PMC7654932; doi:10.1093/eurheartj/ehaa070)
Supplement: ehaa070_supplementary_data [file ehaa070_supplementary_data.docx]

Supplementary materials

**Plasma lipids and risk of aortic valve stenosis: A Mendelian randomization study**

Milad Nazarzadeh, Ana-Catarina Pinho-Gomes, Zeinab Bidel, Abbas Dehghan, Dexter Canoy, Abdelaali Hassaine, Roberto Ayales Solares, Gholamreza Salimi-Khorshidi, George Davey Smith, Catherine M. Otto, Kazem Rahimi

Contents

[Figure S1. Diagram of Mendelian randomization framework in the current paper. 4](#_Toc29464912)

[Figure S2. Predefined decision tree for the selection of methods to evaluate associations using two-sample Mendelian randomization. 5](#_Toc29464913)

[Table S1. Number of valvular heart disease cases and minimum detectable odds ratio in Mendelian randomization study of blood lipid profile and risk of valvular heart disease. 6](#_Toc29464914)

[Text S1. Diagnostic codes for identification of outcomes. 7](#_Toc29464915)

[Aortic valve stenosis 7](#_Toc29464916)

[Aortic valve regurgitation 7](#_Toc29464917)

[Mitral valve regurgitation 7](#_Toc29464918)

[Myocardial infraction (any type) 7](#_Toc29464919)

[Heart Failure 7](#_Toc29464920)

[Aortic valve replacement 8](#_Toc29464921)

[Figure S3. Comparison of the causal estimates from the various Mendelian randomization methods as sensitivity analysis. 9](#_Toc29464922)

[A. HDL cholesterol and aortic stenosis 9](#_Toc29464923)

[B. HDL cholesterol and aortic regurgitation 10](#_Toc29464924)

[C. HDL cholesterol and mitral regurgitation 11](#_Toc29464925)

[D. LDL cholesterol and aortic stenosis 12](#_Toc29464926)

[E. LDL cholesterol and aortic regurgitation 13](#_Toc29464927)

[F. LDL cholesterol and mitral regurgitation 14](#_Toc29464928)

[G. Total cholesterol and aortic stenosis 15](#_Toc29464929)

[H. Total cholesterol and aortic regurgitation 16](#_Toc29464930)

[I. Total cholesterol and mitral regurgitation 17](#_Toc29464931)

[J. Triglycerides and aortic stenosis 18](#_Toc29464932)

[K. Triglycerides and aortic regurgitation 19](#_Toc29464933)

[L. Triglycerides and mitral regurgitation 20](#_Toc29464934)

[Figure S4. Forest plot of variant specific inverse variance estimates for causal association between high-density lipoproteins (HDL) and valvular heart disease. 21](#_Toc29464935)

[Figure S5. Funnel plot of causal association between high-density lipoproteins (HDL) and valvular heart disease. 22](#_Toc29464936)

[Figure S6. Forest plot of variant specific inverse variance estimates for causal association between low-density lipoproteins (LDL) and valvular heart disease. 23](#_Toc29464937)

[Figure S7. Funnel plot of causal association between low-density lipoproteins (LDL) and valvular heart disease. 24](#_Toc29464938)

[Figure S8. Forest plot of variant specific inverse variance estimates for causal association between total cholesterol and valvular heart disease. 25](#_Toc29464939)

[Figure S9. Funnel plot of causal association between total cholesterol and valvular heart disease. 26](#_Toc29464940)

[Figure S10. Forest plot of variant specific inverse variance estimates for causal association between triglyceride and valvular heart disease. 27](#_Toc29464941)

[Figure S11. Funnel plot of causal association between triglyceride and valvular heart disease. 28](#_Toc29464942)

[Table S2. Two sample Mendelian randomization estimations showing the effect of lipid profile on aortic stenosis excluding myocardial infraction cases. 29](#_Toc29464943)

[Table S3. Two sample Mendelian randomization estimations showing the effect of lipid profile on aortic regurgitation after excluding myocardial infraction cases. 30](#_Toc29464944)

[Table S4. Two sample Mendelian randomization estimations showing the effect of lipid profile on mitral regurgitation excluding participants with myocardial infarction. 31](#_Toc29464945)

[Figure S12. Two sample Mendelian randomization estimations showing the effect of lipid profile on coronary heart disease as control outcome 32](#_Toc29464946)

[Figure S13. Two sample Mendelian randomization estimations showing the effect of lipid profile on valvular heart disease after excluding of myocardial infraction cases as sensitivity analysis. 33](#_Toc29464947)

[Figure S14. Two sample Mendelian randomization estimations showing the effect of lipid profile on valvular heart disease after excluding participants with heart failure as sensitivity analysis. 34](#_Toc29464948)

[Figure S15. Sensitivity analysis for assessing the effect of severe cases including aortic valve replacement. 35](#_Toc29464949)

[Figure S16. Leave-one-out plot to assess if a single variant is driving the association between HDL cholesterol and valvular heart disease. 36](#_Toc29464950)

[Figure S17. Leave-one-out plot to assess if a single SNP is driving the association between LDL cholesterol and valvular heart disease. 37](#_Toc29464951)

[Figure S18. Leave-one-out plot to assess if a single SNP is driving the association between total cholesterol and valvular heart disease. 38](#_Toc29464952)

[Figure S19. Leave-one-out plot to assess if a single SNP is driving the association between triglyceride and valvular heart disease. 39](#_Toc29464953)

[Figure S20. Comparison of the direct causal estimations between lipid profile and valvular heart disease risk using Multivariable Mendelian randomization, additionally adjusted for lipoprotein (a). 40](#_Toc29464954)

[Dataset 1. Harmonized dataset of two-sample Mendelian randomization analysis for the effect of HDL cholesterol on valvular heart disease. 41](#_Toc29464955)

[Dataset 2. Harmonized dataset of two-sample Mendelian randomization for the effect of LDL cholesterol on valvular heart disease. 44](#_Toc29464956)

[Dataset 3. Harmonized dataset of two-sample Mendelian randomization for the effect of total cholesterol on valvular heart disease. 47](#_Toc29464957)

[Dataset 4. Harmonized dataset of two-sample Mendelian randomization for the effect of triglyceride on valvular heart disease. 50](#_Toc29464958)

# Figure S1. Diagram of Mendelian randomization framework in the current paper.


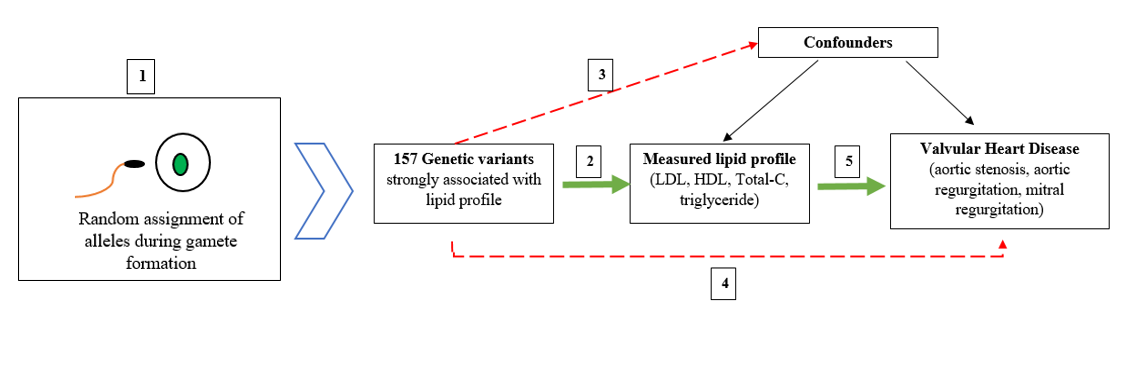


1. A zygote consists of gametes formed from the union of the sperm cell and the ovum. During the formation of the zygote, one allele from father (1/2) and one from mother (1/2) randomly inherit to baby. A well-known example is probability of baby gender (1/2 male and 1/2 female); 2.genetic variants should have strong association with lipid profile measures; 3.genetic variants should not have significant association with confounders; 4.genetic variants should not have any significant association with outcome of interest; 5. If the mentioned assumptions exist, we can investigate causality of association.

# Figure S2. Predefined decision tree for the selection of methods to evaluate associations using two-sample Mendelian randomization.


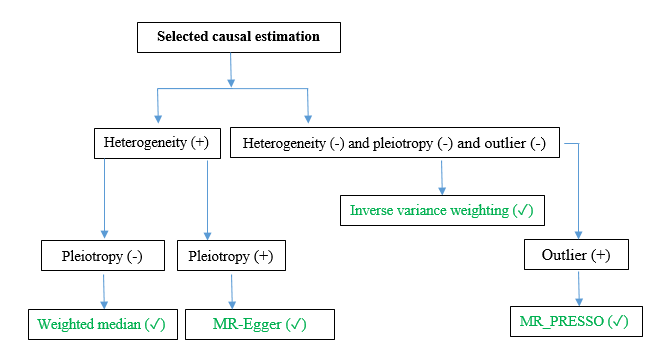


| Table S1. Number of valvular heart disease cases and minimum detectable odds ratio in Mendelian randomization study of blood lipid profile and risk of valvular heart disease. | | | | | | | | | |
| --- | --- | --- | --- | --- | --- | --- | --- | --- | --- |
| Valvular disease type | Cases | Total | Proportion of cases | Minimum detectable odds ratio | | | | | |
|  |  |  |  | r^2^ = 1% | r^2^ = 2% | r^2^=5% | r^2^=10% | r^2^ =12% | r^2^ =15% |
| Aortic stenosis | 1 961 | 432 173 | 0.0045 | 1.64 | 1.45 | 1.29 | 1.20 | 1.18 | 1.16 |
| Aortic regurgitation | 736 | 432 173 | 0.0017 | 2.04 | 1.73 | 1.46 | 1.32 | 1.30 | 1.26 |
| Mitral regurgitation | 2 213 | 432 173 | 0.0051 | 1.60 | 1.42 | 1.26 | 1.18 | 1.17 | 1.15 |
| Minimum detectable odds ratio (OR) per 1-standard deviation increase in lipid profile measures. Assumptions: 80% statistical power, 5% alpha level and 2-15 % of variance is explained by the selected SNPs in this paper ( r^2^). | | | | | | | | | |

Considering a sample size of 432 173 participants and 10% variance explanation by selected SNPs, the current Mendelian randomization analysis had 80% statistical power to detect a minimum causal effect per 1-SD increase of any of the plasma lipids parameters of 20% (OR=1.20) for aortic stenosis, 32% (OR=1.32) for aortic regurgitation, and 18% (OR=1.18) for mitral regurgitation. Estimated minimum detectable OR for different scenarios of variance explained by selected SNPs are provided in Table S1.

# Text S1. Diagnostic codes for identification of outcomes.

## Aortic valve stenosis

1. **Aortic valve stenosis identified from linkage of UK Biobank cohort to hospitalisation database (Hospital Episode Statistics)**: One (or more) of the following aortic valve stenosis codes in HES, SMR01 or PEDW linked records in the primary or any secondary position: ICD 10 codes: I352, I350
2. **Aortic valve stenosis based on UK Biobank participant self-reports:** participant recorded as having aortic valve stenosis by self-report on the basis of verbal interview with research nurse (regardless of response to the direct touchscreen question about MI), but without evidence of aortic valve stenosis from linked HES, SMR01 or PEDW data (as defined above). UK Biobank field 20002, codes: 1490

## Aortic valve regurgitation

1. **Aortic valve regurgitation identified from hospitalisation database**: ICD 10 codes: I351
2. **Aortic valve regurgitation by self-report:** UK Biobank field 20002, codes: 1587

## Mitral valve regurgitation

1. **Mitral valve regurgitation identified from hospitalisation database**: ICD 10 codes: I340
2. **Mitral valve regurgitation by self-report:** UK Biobank field 20002, codes: NA

## Myocardial infraction (any type)

1. **Myocardial infraction (MI) identified from hospitalisation database**:

ICD 9 codes: 4109, 4119, 4129

ICD 10 codes: I241, I252, I210, I211, I212, I213, I214, I219, I220, I221, I228, I229, I231, I236, I238

1. **MI by self-report**: UK Biobank field 20002, codes: 1075

## Heart Failure

1. **Heart failure identified from hospitalisation database**:

ICD9 codes: 4280, 4281

ICD 10 codes: I500, I501, I509

1. **Heart failure by self-report**: UK Biobank field 20002, codes: 1076

| Aortic valve replacement |
| --- |
| **1.Operative procedures - main OPCS** **codes** ([OPCS Classification of Interventions and Procedures](https://www.datadictionary.nhs.uk/web_site_content/supporting_information/clinical_coding/opcs_classification_of_interventions_and_procedures.asp)) |
| **K26 Plastic repair of aortic valve** |
| - K26.1 Allograft replacement of aortic valve |
| - K26.2 Xenograft replacement of aortic valve |
| - K26.3 Prosthetic replacement of aortic valve |
| - K26.4 Replacement of aortic valve NEC |
| - K26.5 Aortic valve repair NEC |
| - K26.8 Other specified plastic repair of aortic valve |
| - K26.9 Unspecified plastic repair of aortic valve |
| **2.Operative procedures - secondary OPCS** |
| **K26 Plastic repair of aortic valve** |
| - K26.1 Allograft replacement of aortic valve |
| - K26.2 Xenograft replacement of aortic valve |
| - K26.3 Prosthetic replacement of aortic valve |
| - K26.4 Replacement of aortic valve NEC |
| - K26.5 Aortic valve repair NEC |
| - K26.8 Other specified plastic repair of aortic valve |

# Figure S3. Comparison of the causal estimates from the various Mendelian randomization methods as sensitivity analysis.

## HDL cholesterol and aortic stenosis

| Methods | OR | CI 95% | | P-value |
| --- | --- | --- | --- | --- |
| Simple median | 0.77 | 0.54 | 1.11 | 0.17 |
| Weighted median | 0.99 | 0.76 | 1.29 | 0.93 |
| Penalized weighted median | 1.00 | 0.76 | 1.30 | 0.97 |
| Inverse variance weighting (IVW) | 0.86 | 0.69 | 1.07 | 0.18 |
| Penalized IVW | 0.86 | 0.70 | 1.05 | 0.14 |
| Robust IVW | 0.88 | 0.69 | 1.12 | 0.30 |
| Penalized robust IVW | 0.88 | 0.69 | 1.12 | 0.29 |
| MR-Egger | 0.98 | 0.70 | 1.37 | 0.91 |
| Penalized MR-Egger | 1.02 | 0.75 | 1.39 | 0.91 |
| Robust MR-Egger | 1.02 | 0.79 | 1.30 | 0.90 |
| Penalized robust MR-Egger | 1.03 | 0.84 | 1.28 | 0.76 |


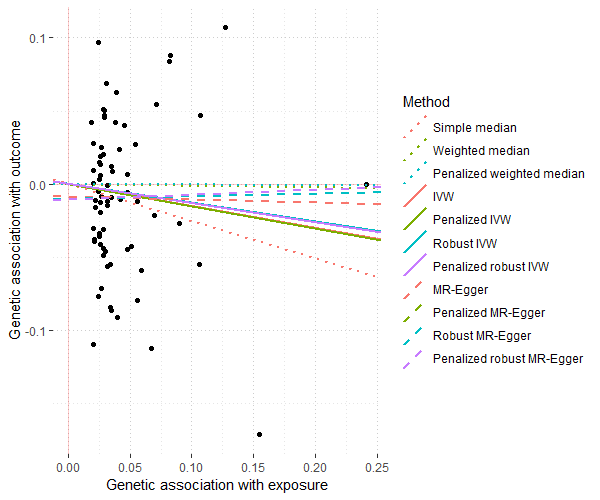


## HDL cholesterol and aortic regurgitation

| Methods | OR | CI 95% | | P-value |
| --- | --- | --- | --- | --- |
| Simple median | 0.73 | 0.43 | 1.23 | 0.23 |
| Weighted median | 0.73 | 0.47 | 1.13 | 0.15 |
| Penalized weighted median | 0.73 | 0.47 | 1.12 | 0.15 |
| Inverse variance weighting (IVW) | 0.88 | 0.67 | 1.16 | 0.35 |
| Penalized IVW | 0.88 | 0.67 | 1.16 | 0.35 |
| Robust IVW | 0.84 | 0.61 | 1.15 | 0.28 |
| Penalized robust IVW | 0.84 | 0.61 | 1.15 | 0.28 |
| MR-Egger | 0.82 | 0.53 | 1.25 | 0.35 |
| Penalized MR-Egger | 0.82 | 0.53 | 1.25 | 0.35 |
| Robust MR-Egger | 0.78 | 0.54 | 1.12 | 0.17 |
| Penalized robust MR-Egger | 0.78 | 0.54 | 1.12 | 0.17 |


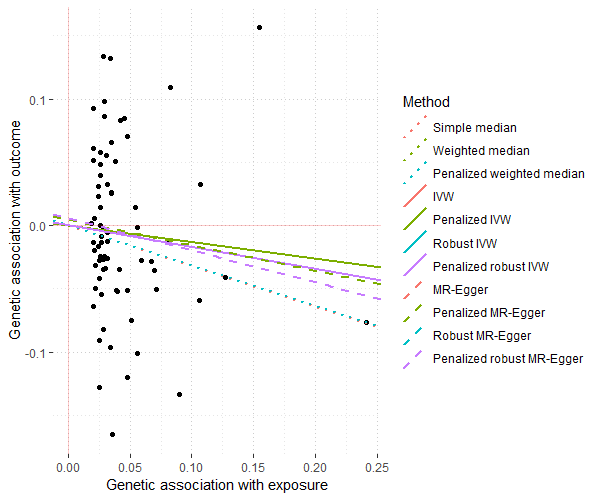


## HDL cholesterol and mitral regurgitation


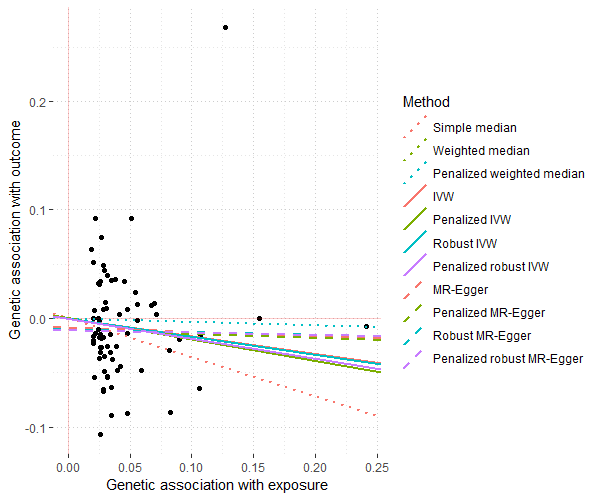


| Methods | OR | CI 95% | | P-value |
| --- | --- | --- | --- | --- |
| Simple median | 0.70 | 0.51 | 0.95 | 0.02 |
| Weighted median | 0.97 | 0.76 | 1.23 | 0.79 |
| Penalized weighted median | 0.97 | 0.76 | 1.23 | 0.79 |
| Inverse variance weighting (IVW) | 0.85 | 0.70 | 1.02 | 0.09 |
| Penalized IVW | 0.82 | 0.69 | 0.98 | 0.03 |
| Robust IVW | 0.85 | 0.71 | 1.00 | 0.05 |
| Penalized robust IVW | 0.83 | 0.70 | 0.99 | 0.04 |
| MR-Egger | 0.96 | 0.72 | 1.29 | 0.80 |
| Penalized MR-Egger | 0.96 | 0.73 | 1.26 | 0.78 |
| Robust MR-Egger | 0.98 | 0.85 | 1.13 | 0.75 |
| Penalized robust MR-Egger | 0.98 | 0.85 | 1.12 | 0.75 |

## LDL cholesterol and aortic stenosis


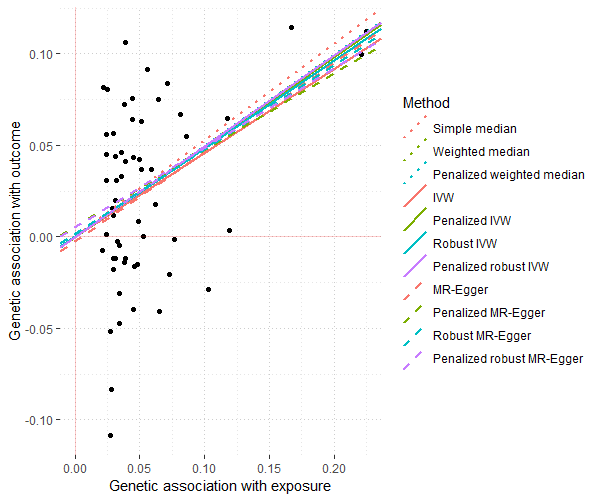


| Methods | OR | CI 95% | | P-value |
| --- | --- | --- | --- | --- |
| Simple median | 1.70 | 1.26 | 2.29 | 5.44E-04 |
| Weighted median | 1.64 | 1.28 | 2.11 | 1.07E-04 |
| Penalized weighted median | 1.64 | 1.28 | 2.11 | 1.01E-04 |
| Inverse variance weighting (IVW) | 1.58 | 1.30 | 1.92 | 3.15E-06 |
| Penalized IVW | 1.64 | 1.38 | 1.93 | 7.16E-09 |
| Robust IVW | 1.62 | 1.38 | 1.90 | 2.05E-09 |
| Penalized robust IVW | 1.64 | 1.41 | 1.91 | 1.77E-10 |
| MR-Egger | 1.63 | 1.19 | 2.24 | 2.38E-03 |
| Penalized MR-Egger | 1.52 | 1.15 | 2.00 | 2.84E-03 |
| Robust MR-Egger | 1.59 | 1.29 | 1.95 | 1.19E-05 |
| Penalized robust MR-Egger | 1.54 | 1.26 | 1.89 | 2.34E-05 |

## LDL cholesterol and aortic regurgitation


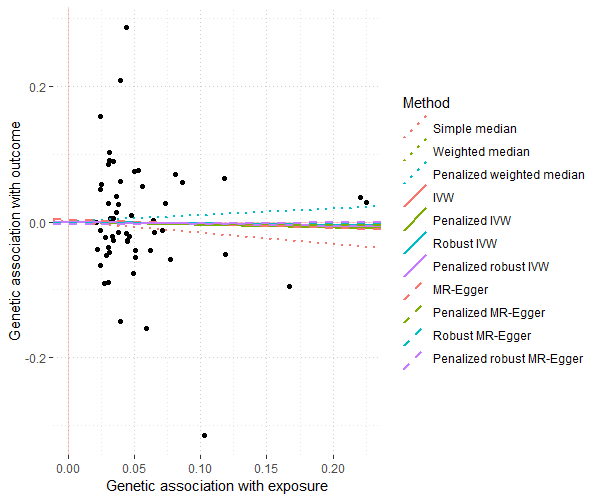


| Methods | OR | CI 95% | | P-value |
| --- | --- | --- | --- | --- |
| Simple median | 0.85 | 0.53 | 1.36 | 0.49 |
| Weighted median | 1.10 | 0.73 | 1.66 | 0.63 |
| Penalized weighted median | 1.10 | 0.73 | 1.66 | 0.63 |
| Inverse variance weighting (IVW) | 0.98 | 0.73 | 1.31 | 0.88 |
| Penalized IVW | 0.96 | 0.73 | 1.26 | 0.77 |
| Robust IVW | 0.98 | 0.76 | 1.26 | 0.86 |
| Penalized robust IVW | 0.97 | 0.75 | 1.25 | 0.82 |
| MR-Egger | 0.94 | 0.59 | 1.51 | 0.80 |
| Penalized MR-Egger | 0.99 | 0.63 | 1.55 | 0.95 |
| Robust MR-Egger | 1.00 | 0.66 | 1.52 | 0.99 |
| Penalized robust MR-Egger | 1.02 | 0.68 | 1.51 | 0.94 |

## LDL cholesterol and mitral regurgitation


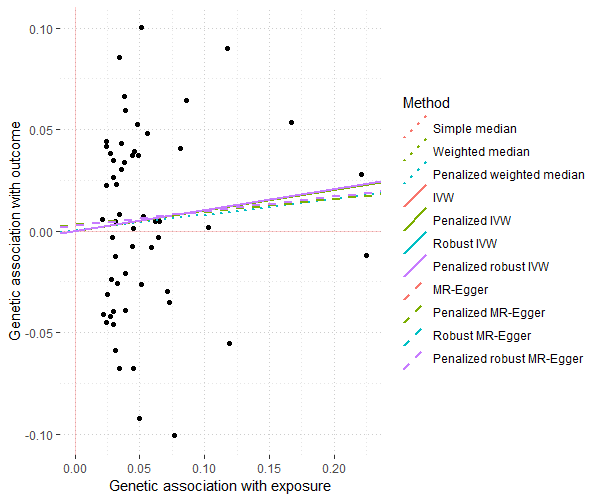


| Methods | OR | CI 95% | | P-value |
| --- | --- | --- | --- | --- |
| Simple median | 1.08 | 0.81 | 1.44 | 0.59 |
| Weighted median | 1.08 | 0.85 | 1.37 | 0.52 |
| Penalized weighted median | 1.08 | 0.85 | 1.37 | 0.51 |
| Inverse variance weighting (IVW) | 1.11 | 0.94 | 1.31 | 0.24 |
| Penalized IVW | 1.11 | 0.94 | 1.31 | 0.24 |
| Robust IVW | 1.11 | 0.93 | 1.32 | 0.24 |
| Penalized robust IVW | 1.11 | 0.93 | 1.32 | 0.24 |
| MR-Egger | 1.07 | 0.81 | 1.40 | 0.65 |
| Penalized MR-Egger | 1.00 | 0.99 | 1.02 | 0.74 |
| Robust MR-Egger | 1.07 | 0.81 | 1.40 | 0.65 |
| Penalized robust MR-Egger | 1.00 | 0.99 | 1.02 | 0.74 |

| Methods | OR | CI 95% | | P-value |
| --- | --- | --- | --- | --- |
| Simple median | 1.61 | 1.20 | 2.14 | 1.31E-03 |
| Weighted median | 1.73 | 1.33 | 2.25 | 3.74E-05 |
| Penalized weighted median | 1.73 | 1.33 | 2.25 | 3.60E-05 |
| Inverse variance weighting (IVW) | 1.60 | 1.33 | 1.92 | 4.08E-07 |
| Penalized IVW | 1.66 | 1.41 | 1.95 | 1.03E-09 |
| Robust IVW | 1.65 | 1.39 | 1.96 | 8.51E-09 |
| Penalized robust IVW | 1.67 | 1.41 | 1.96 | 1.33E-09 |
| MR-Egger | 1.82 | 1.32 | 2.53 | 3.00E-04 |
| Penalized MR-Egger | 1.73 | 1.29 | 2.33 | 2.76E-04 |
| Robust MR-Egger | 1.76 | 1.43 | 2.17 | 1.51E-07 |
| Penalized robust MR-Egger | 1.74 | 1.41 | 2.14 | 1.85E-07 |

## Total cholesterol and aortic stenosis


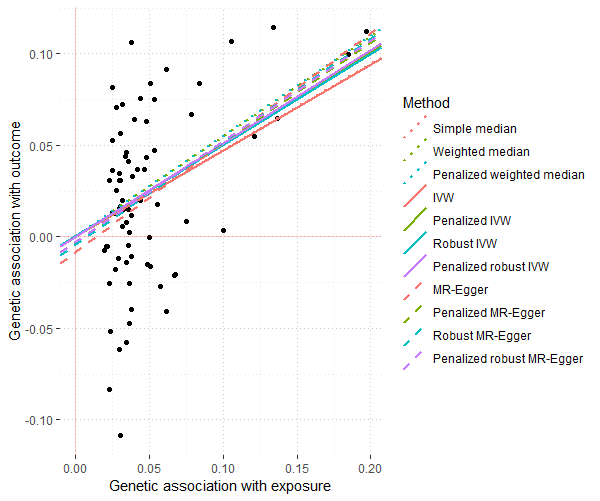


## Total cholesterol and aortic regurgitation


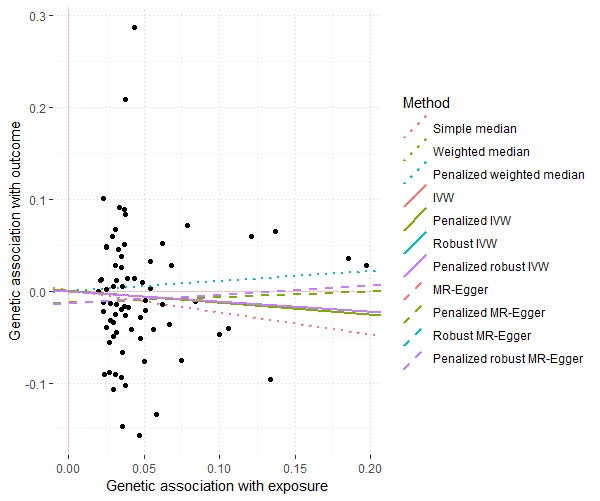


| Methods | OR | CI 95% | | P-value |
| --- | --- | --- | --- | --- |
| Simple median | 0.79 | 0.50 | 1.24 | 0.30 |
| Weighted median | 1.11 | 0.74 | 1.69 | 0.61 |
| Penalized weighted median | 1.12 | 0.74 | 1.69 | 0.60 |
| Inverse variance weighting (IVW) | 0.88 | 0.68 | 1.14 | 0.33 |
| Penalized IVW | 0.88 | 0.68 | 1.14 | 0.33 |
| Robust IVW | 0.89 | 0.67 | 1.20 | 0.45 |
| Penalized robust IVW | 0.89 | 0.67 | 1.20 | 0.45 |
| MR-Egger | 1.06 | 0.67 | 1.69 | 0.80 |
| Penalized MR-Egger | 1.06 | 0.67 | 1.69 | 0.80 |
| Robust MR-Egger | 1.10 | 0.72 | 1.68 | 0.66 |
| Penalized robust MR-Egger | 1.10 | 0.72 | 1.68 | 0.66 |

## Total cholesterol and mitral regurgitation


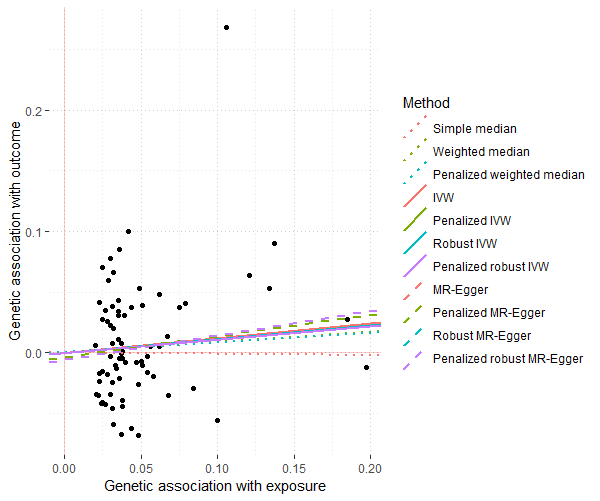


| Methods | OR | CI 95% | | P-value |
| --- | --- | --- | --- | --- |
| Simple median | 0.99 | 0.75 | 1.29 | 0.94 |
| Weighted median | 1.08 | 0.85 | 1.39 | 0.50 |
| Penalized weighted median | 1.08 | 0.84 | 1.38 | 0.51 |
| Inverse variance weighting (IVW) | 1.12 | 0.95 | 1.32 | 0.14 |
| Penalized IVW | 1.12 | 0.95 | 1.31 | 0.16 |
| Robust IVW | 1.12 | 0.94 | 1.32 | 0.19 |
| Penalized robust IVW | 1.11 | 0.94 | 1.32 | 0.20 |
| MR-Egger | 1.19 | 0.88 | 1.60 | 0.24 |
| Penalized MR-Egger | 1.19 | 0.88 | 1.60 | 0.243 |
| Robust MR-Egger | 1.22 | 0.87 | 1.72 | 0.24 |
| Penalized robust MR-Egger | 1.22 | 0.87 | 1.72 | 0.24 |

## Triglycerides and aortic stenosis


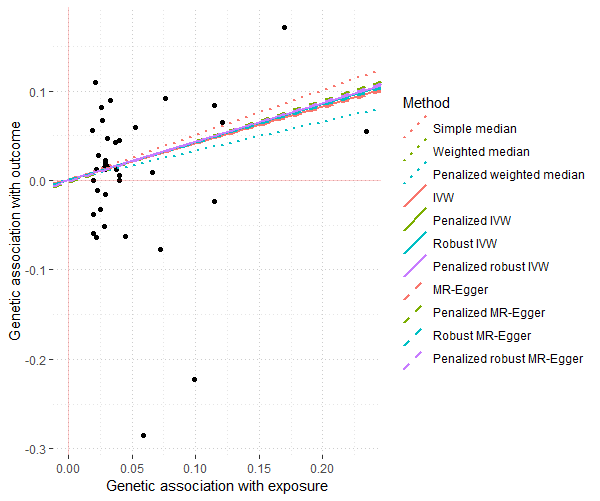


| Methods | OR | CI 95% | | P-value |
| --- | --- | --- | --- | --- |
| Simple median | 1.66 | 1.09 | 2.53 | 0.02 |
| Weighted median | 1.39 | 1.00 | 1.92 | 0.05 |
| Penalized weighted median | 1.39 | 1.00 | 1.92 | 0.05 |
| Inverse variance weighting (IVW) | 1.52 | 1.13 | 2.03 | 0.01 |
| Penalized IVW | 1.53 | 1.19 | 1.97 | 0.00 |
| Robust IVW | 1.53 | 1.15 | 2.04 | 0.00 |
| Penalized robust IVW | 1.54 | 1.15 | 2.05 | 0.00 |
| MR-Egger | 1.49 | 0.95 | 2.33 | 0.08 |
| Penalized MR-Egger | 1.58 | 1.08 | 2.33 | 0.02 |
| Robust MR-Egger | 1.51 | 0.98 | 2.33 | 0.06 |
| Penalized robust MR-Egger | 1.57 | 1.01 | 2.44 | 0.05 |

## Triglycerides and aortic regurgitation

| Methods | OR | CI 95% | | P-value |
| --- | --- | --- | --- | --- |
| Simple median | 1.31 | 0.71 | 2.43 | 0.39 |
| Weighted median | 1.26 | 0.77 | 2.06 | 0.36 |
| Penalized weighted median | 1.29 | 0.77 | 2.14 | 0.33 |
| Inverse variance weighting (IVW) | 1.01 | 0.70 | 1.46 | 0.95 |
| Penalized IVW | 1.04 | 0.75 | 1.46 | 0.80 |
| Robust IVW | 1.11 | 0.76 | 1.63 | 0.58 |
| Penalized robust IVW | 1.12 | 0.77 | 1.63 | 0.54 |
| MR-Egger | 1.21 | 0.70 | 2.09 | 0.50 |
| Penalized MR-Egger | 1.15 | 0.69 | 1.93 | 0.59 |
| Robust MR-Egger | 1.24 | 0.78 | 1.98 | 0.37 |
| Penalized robust MR-Egger | 1.23 | 0.77 | 1.97 | 0.39 |


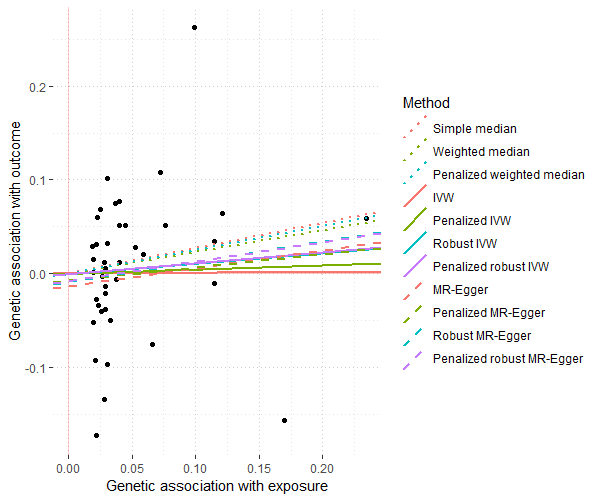


##
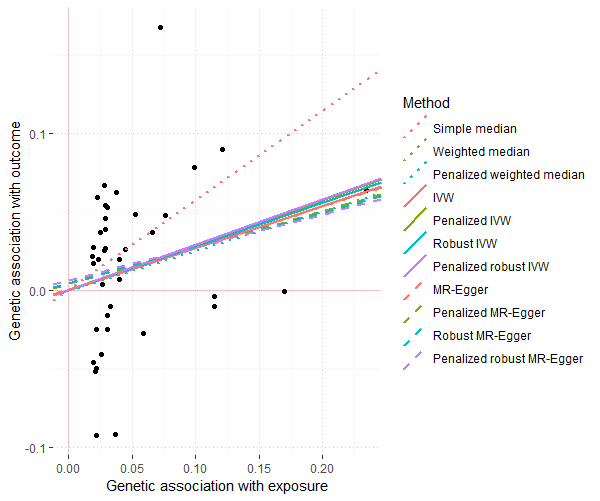
Triglycerides and mitral regurgitation

| Methods | OR | CI 95% | | P-value |
| --- | --- | --- | --- | --- |
| Simple median | 1.77 | 1.23 | 2.55 | 0.0020 |
| Weighted median | 1.29 | 0.96 | 1.73 | 0.0936 |
| Penalized weighted median | 1.29 | 0.96 | 1.73 | 0.0939 |
| Inverse variance weighting (IVW) | 1.31 | 1.04 | 1.65 | 0.0202 |
| Penalized IVW | 1.33 | 1.08 | 1.65 | 0.0075 |
| Robust IVW | 1.33 | 1.10 | 1.59 | 0.0029 |
| Penalized robust IVW | 1.34 | 1.12 | 1.61 | 0.0017 |
| MR-Egger | 1.30 | 0.92 | 1.85 | 0.1356 |
| Penalized MR-Egger | 1.26 | 0.91 | 1.75 | 0.1597 |
| Robust MR-Egger | 1.25 | 0.97 | 1.62 | 0.0884 |
| Penalized robust MR-Egger | 1.24 | 0.97 | 1.58 | 0.0930 |

# Figure S4. Forest plot of variant specific inverse variance estimates for causal association between high-density lipoproteins (HDL) and valvular heart disease.

IVW: inverse variance waiting;

**Mitral regurgitation**

**Aortic regurgitation**

**Aortic stenosis**

# Figure S5. Funnel plot of causal association between high-density lipoproteins (HDL) and valvular heart disease.

**Mitral regurgitation**

**Aortic regurgitation**

**Aortic stenosis**

# Figure S6. Forest plot of variant specific inverse variance estimates for causal association between low-density lipoproteins (LDL) and valvular heart disease.

**Mitral regurgitation**

**Aortic regurgitation**

**Aortic stenosis**

# Figure S7. Funnel plot of causal association between low-density lipoproteins (LDL) and valvular heart disease.

**Mitral regurgitation**

**Aortic regurgitation**

**Aortic stenosis**

# Figure S8. Forest plot of variant specific inverse variance estimates for causal association between total cholesterol and valvular heart disease.

**Aortic stenosis**

**Mitral regurgitation**

**Aortic regurgitation**

# Figure S9. Funnel plot of causal association between total cholesterol and valvular heart disease.

**Mitral regurgitation**

**Aortic regurgitation**

**Aortic stenosis**

# Figure S10. Forest plot of variant specific inverse variance estimates for causal association between triglyceride and valvular heart disease.

**Aortic stenosis**

**Aortic regurgitation**

**Mitral regurgitation**

# Figure S11. Funnel plot of causal association between triglyceride and valvular heart disease.

**Aortic regurgitation**

**Aortic stenosis**

**Mitral regurgitation**

| Table S2. Two sample Mendelian randomization estimations showing the effect of lipid profile on aortic stenosis excluding myocardial infraction cases. | | | | | | | |
| --- | --- | --- | --- | --- | --- | --- | --- |
| Methods | Exposure | Odds ratio ǂ | 95% CI | | P-value | P_h_ | Q-statistics |
| Inverse-variance weighted | HDL | 0.84 | 0.66 | 1.07 | 0.16 | < 0.001 | 113.1 |
| MR-Egger |  | 0.98 | 0.67 | 1.41 | 0.91 |  |  |
| Weighted Median |  | 0.90 | 0.67 | 1.22 | 0.52 |  |  |
| MR-Egger intercept * |  | -0.01 | -0.02 | 0.008 | 0.29 |  |  |
| Inverse-variance weighted | LDL | 1.60 | 1.30 | 1.96 | < 0.001 | 0.052 | 74.1 |
| MR-Egger |  | 1.54 | 1.10 | 2.16 | < 0.01 |  |  |
| Weighted Median |  | 1.59 | 1.22 | 2.07 | < 0.001 |  |  |
| MR-Egger intercept * |  | 0.002 | -0.018 | 0.023 | 0.79 |  |  |
| Inverse-variance weighted | Total cholesterol | 1.59 | 1.30 | 1.94 | < 0.001 | 0.07 | 90.4 |
| MR-Egger |  | 1.72 | 1.20 | 2.47 | 0.002 |  |  |
| Weighted Median |  | 1.67 | 1.26 | 2.20 | < 0.001 |  |  |
| MR-Egger intercept * |  | - 0.005 | -0.024 | 0.014 | 0.59 |  |  |
| Inverse-variance weighted | Triglyceride | 1.57 | 1.16 | 2.14 | 0.003 | 0.002 | 68.3 |
| MR-Egger |  | 1.46 | 0.92 | 2.33 | 0.11 |  |  |
| Weighted Median |  | 1.50 | 1.06 | 2.10 | 0.02 |  |  |
| MR-Egger intercept * |  | 0.005 | -0.020 | 0.031 | 0.67 |  |  |
| HDL: high density lipoprotein; LDL: Low density lipoprotein; CI: confidence intervals; P_h_: P-value for heterogeneity  * Regression coefficient (95% CI)  ǂ Odds ratio per 1-standard deviation increase in lipid profile measures | | | | | | | |

| Table S3. Two sample Mendelian randomization estimations showing the effect of lipid profile on aortic regurgitation after excluding myocardial infraction cases. | | | | | | | |
| --- | --- | --- | --- | --- | --- | --- | --- |
| **Methods** | Exposure | Odds ratio ǂ | 95% CI | | P-value | P_h_ | Q-statistics |
| Inverse-variance weighted | HDL | 0.88 | 0.65 | 1.19 | 0.39 | 0.68 | 63.9 |
| MR-Egger |  | 0.73 | 0.46 | 1.16 | 0.17 |  |  |
| Weighted Median |  | 0.74 | 0.46 | 1.19 | 0.21 |  |  |
| MR-Egger intercept* |  | 0.012 | -0.011 | 0.036 | 0.29 |  |  |
| Inverse-variance weighted | LDL | 0.89 | 0.65 | 1.23 | 0.48 | 0.09 | 70.6 |
| MR-Egger |  | 0.97 | 0.57 | 1.64 | 0.90 |  |  |
| Weighted Median |  | 0.91 | 0.56 | 1.48 | 0.70 |  |  |
| MR-Egger intercept* |  | -0.006 | -0.039 | 0.026 | 0.69 |  |  |
| Inverse-variance weighted | Total cholesterol | 0.87 | 0.65 | 1.16 | 0.33 | 0.51 | 70.7 |
| MR-Egger |  | 1.04 | 0.62 | 1.73 | 0.89 |  |  |
| Weighted Median |  | 0.90 | 0.55 | 1.48 | 0.66 |  |  |
| MR-Egger intercept* |  | -0.011 | -0.039 | 0.016 | 0.42 |  |  |
| Inverse-variance weighted | Triglyceride | 1.18 | 0.80 | 1.76 | 0.40 | 0.22 | 45.2 |
| MR-Egger |  | 1.53 | 0.84 | 2.77 | 0.16 |  |  |
| Weighted Median |  | 1.25 | 0.74 | 2.12 | 0.40 |  |  |
| MR-Egger intercept* |  | - 0.018 | -0.050 | 0.013 | 0.26 |  |  |
| HDL: high density lipoprotein; LDL: Low density lipoprotein; CI: confidence intervals; P_h_: P-value for heterogeneity  * Regression coefficient (95% CI)  ǂ Odds ratio per 1-standard deviation increase in lipid profile measures | | | | | | | |

| Table S4. Two sample Mendelian randomization estimations showing the effect of lipid profile on mitral regurgitation excluding participants with myocardial infarction. | | | | | | | |
| --- | --- | --- | --- | --- | --- | --- | --- |
| **Methods** | Exposure | Odds ratio ǂ | 95% CI | | P-value | P_h_ | Q statistics |
| Inverse-variance weighted | HDL | 0.91 | 0.73 | 1.12 | 0.37 | 0.02 | 95.4 |
| MR-Egger |  | 0.87 | 0.63 | 1.21 | 0.80 |  |  |
| Weighted Median |  | 1.00 | 0.75 | 1.32 | 0.97 |  |  |
| MR-Egger intercept* |  | 0.002 | -0.014 | 0.019 | 0.76 |  |  |
| Inverse-variance weighted | LDL | 1.04 | 0.84 | 1.28 | 0.72 | 0.01 | 82.4 |
| MR-Egger |  | 0.99 | 0.70 | 1.40 | 0.95 |  |  |
| Weighted Median |  | 0.97 | 0.73 | 1.30 | 0.84 |  |  |
| MR-Egger intercept* |  | 0.003 | -0.017 | 0.025 | 0.73 |  |  |
| Inverse-variance weighted | Total cholesterol | 1.08 | 0.88 | 1.32 | 0.46 | 0.02 | 98.5 |
| MR-Egger |  | 1.06 | 0.74 | 1.54 | 0.74 |  |  |
| Weighted Median |  | 0.97 | 0.72 | 1.30 | 0.83 |  |  |
| MR-Egger intercept* |  | 0.0009 | -0.0189 | 0.0208 | 0.92 |  |  |
| Inverse-variance weighted | Triglyceride | 1.22 | 0.95 | 1.56 | 0.11 | 0.15 | 47.8 |
| MR-Egger |  | 1.38 | 0.95 | 2.02 | 0.09 |  |  |
| Weighted Median |  | 1.26 | 0.90 | 1.76 | 0.17 |  |  |
| MR-Egger intercept* |  | -0.009 | -0.030 | 0.011 | 0.38 |  |  |
| HDL: high density lipoprotein; LDL: Low density lipoprotein; CI: confidence intervals; P_h_: P-value for heterogeneity  * Regression coefficient (95% CI)  ǂ Odds ratio per 1-standard deviation increase in lipid profile measures | | | | | | | |

# Figure S12. Two sample Mendelian randomization estimations showing the effect of lipid profile on coronary heart disease as control outcome


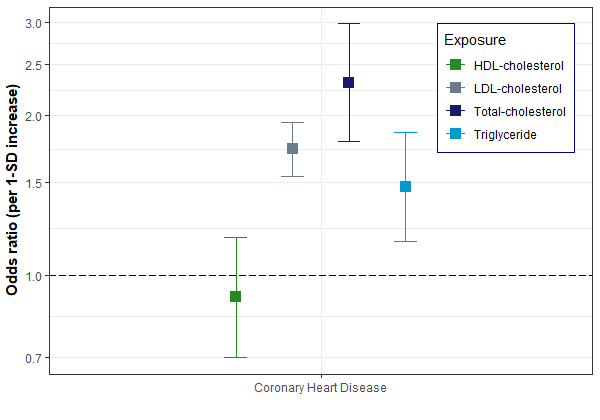


# Figure S13. Two sample Mendelian randomization estimations showing the effect of lipid profile on valvular heart disease after excluding of myocardial infraction cases as sensitivity analysis.


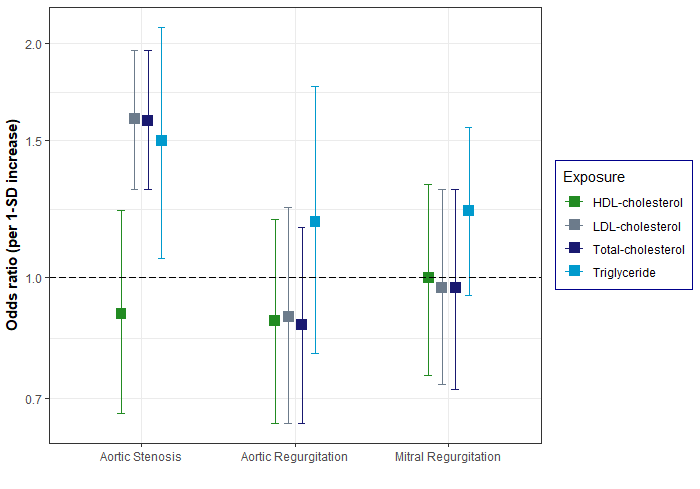


# Figure S14. Two sample Mendelian randomization estimations showing the effect of lipid profile on valvular heart disease after excluding participants with heart failure as sensitivity analysis.


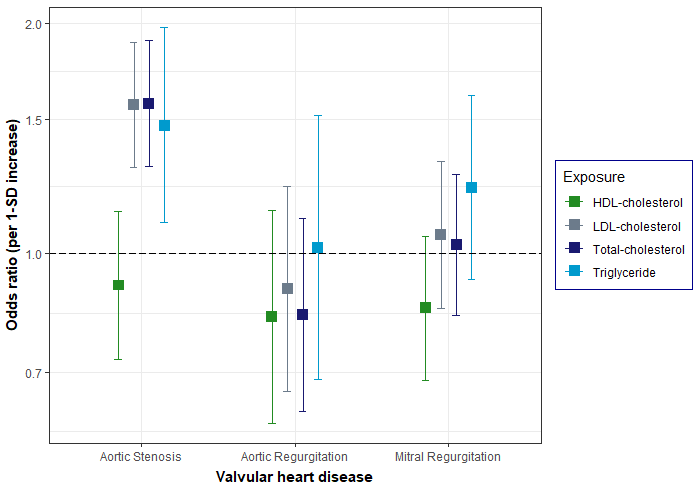


# Figure S15. Sensitivity analysis for assessing the effect of severe cases including aortic valve replacement.

(Number of aortic stenosis cases: 1961; number of aortic stenosis AND aortic replacement therapy case: 957).


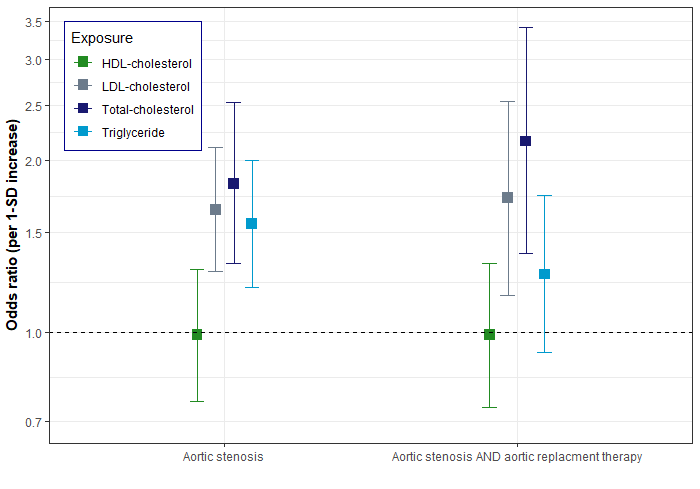


Note: The data for aortic valve replacement was available, but detailed data about the cause of surgery (stenosis or regurgitation) was not available. Although we understand the rationale for using valve replacement surgery as a proxy for more severe valve disease, it is possible that patients may undergo valve replacement for reasons other than the severity of the disease, such as if these patients are undergoing coronary artery bypass grafting. We therefore decided not to consider valve replacement surgery as equivalent to severe valve disease as it could be an erroneous assumption. However, we performed sensitivity analysis with inclusion of aortic valve replacement surgery as outcome and this was in keeping with the main results.

# Figure S16. Leave-one-out plot to assess if a single variant is driving the association between HDL cholesterol and valvular heart disease.

**Mitral regurgitation**

**Aortic regurgitation**


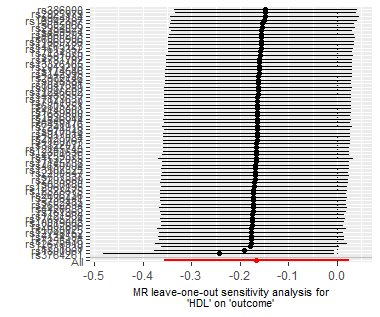

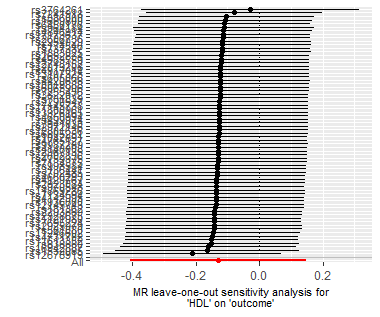

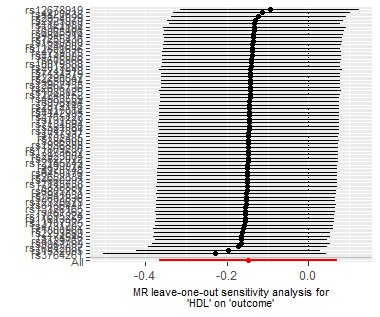


**Aortic stenosis**

# Figure S17. Leave-one-out plot to assess if a single SNP is driving the association between LDL cholesterol and valvular heart disease.

**Aortic stenosis**

**Mitral regurgitation**

**Aortic regurgitation**


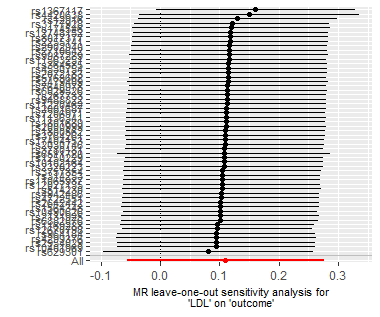

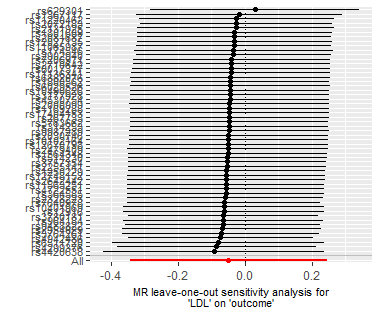

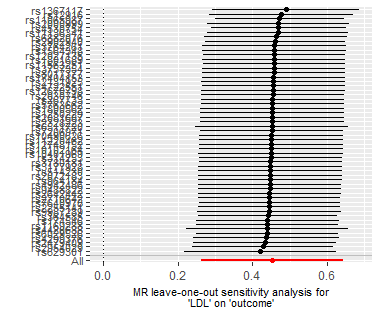


# Figure S18. Leave-one-out plot to assess if a single SNP is driving the association between total cholesterol and valvular heart disease.


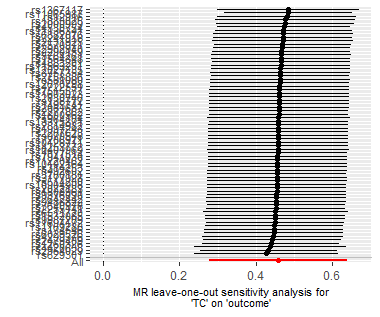


**Mitral regurgitation**

**Aortic regurgitation**

**Aortic stenosis**


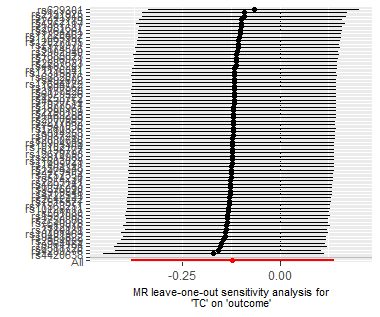

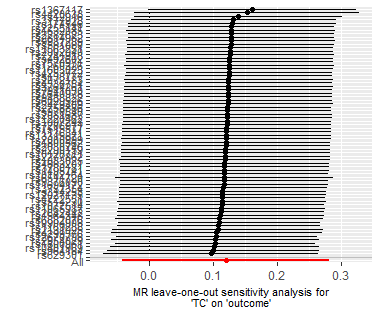


# Figure S19. Leave-one-out plot to assess if a single SNP is driving the association between triglyceride and valvular heart disease.

**Aortic stenosis**

**Mitral regurgitation**

**Aortic regurgitation**


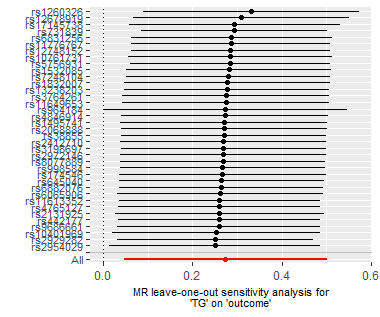

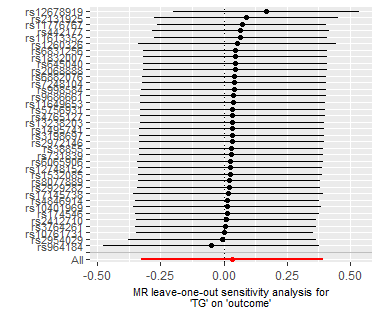

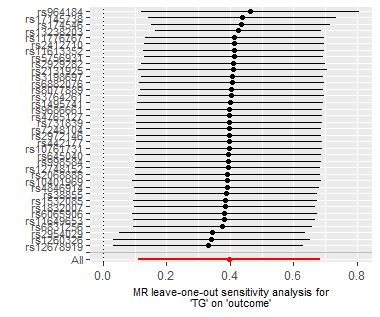


# Figure S20. Comparison of the direct causal estimations between lipid profile and valvular heart disease risk using Multivariable Mendelian randomization, additionally adjusted for lipoprotein (a).

1-SD is equal to 0.98 mmol/L for LDL-cholesterol, 0.41 mmol/L for HDL-cholesterol, 1 mmol/L for triglycerides and 1.10 mmol/L for total cholesterol.


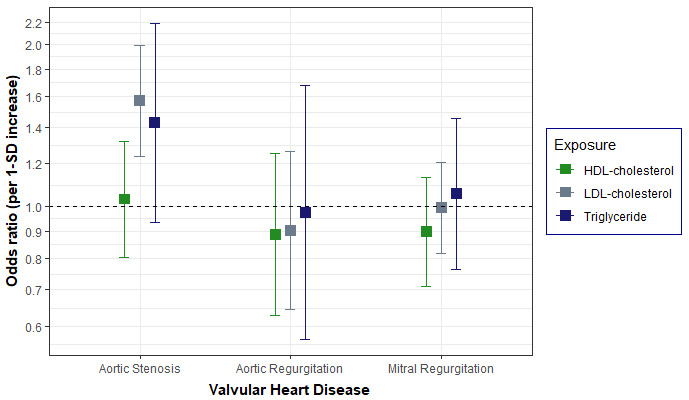


| Dataset 1. Harmonized dataset of two-sample Mendelian randomization analysis for the effect of HDL cholesterol on valvular heart disease. | | | | | | | | | | | | | | |
| --- | --- | --- | --- | --- | --- | --- | --- | --- | --- | --- | --- | --- | --- | --- |
| SNP | Chr | Trait | beta_HDL | se_HDL | effect_allele_HDL | other_allele  _HDL | effect_allele_outcome | other_allele_outcome | beta_AS | se_AS | beta_AR | se_AR | beta_MR | se_MR |
| rs4660293 | 1 | HDL | 0.0350 | 0.00400 | G | A | G | A | 0.012348 | 0.038158 | 0.026207 | 0.062243 | -0.063314 | 0.035160 |
| rs1689800 | 1 | HDL | 0.0340 | 0.00360 | G | A | G | A | -0.054568 | 0.033234 | -0.096674 | 0.053765 | -0.014353 | 0.031411 |
| rs4846914 | 1 | HDL | 0.0480 | 0.00340 | G | A | G | A | -0.044735 | 0.032697 | -0.051682 | 0.053177 | -0.013785 | 0.030860 |
| rs12748152 | 1 | HDL | 0.0510 | 0.00620 | T | C | T | C | -0.042518 | 0.057920 | -0.074969 | 0.093064 | 0.091972 | 0.057652 |
| rs12145743 | 1 | HDL | 0.0200 | 0.00360 | T | G | T | G | 0.009263 | 0.033905 | 0.060970 | 0.054724 | 0.000394 | 0.031929 |
| rs4650994 | 1 | HDL | 0.0210 | 0.00340 | A | G | A | G | -0.037984 | 0.032130 | -0.019316 | 0.052257 | 0.007070 | 0.030175 |
| rs12328675 | 2 | HDL | 0.0450 | 0.00520 | T | C | T | C | 0.040412 | 0.048699 | 0.084414 | 0.077909 | 0.033774 | 0.045943 |
| rs2972146 | 2 | HDL | 0.0320 | 0.00350 | T | G | T | G | -0.014760 | 0.033668 | -0.012395 | 0.054774 | -0.025406 | 0.031726 |
| rs1047891 | 2 | HDL | 0.0270 | 0.00390 | A | C | A | C | -0.008078 | 0.034519 | -0.008253 | 0.056159 | -0.027011 | 0.032367 |
| rs2606736 | 3 | HDL | 0.0250 | 0.00430 | T | C | T | C | -0.033918 | 0.033485 | -0.091101 | 0.054943 | 0.031395 | 0.031254 |
| rs2290547 | 3 | HDL | 0.0300 | 0.00460 | A | G | A | G | -0.045705 | 0.041609 | -0.033718 | 0.067986 | 0.015182 | 0.039882 |
| rs2013208 | 3 | HDL | 0.0250 | 0.00360 | C | T | C | T | -0.035857 | 0.032210 | -0.041434 | 0.052414 | -0.036724 | 0.030298 |
| rs13326165 | 3 | HDL | 0.0290 | 0.00430 | G | A | G | A | 0.045644 | 0.039119 | -0.024646 | 0.064989 | -0.048848 | 0.037866 |
| rs6805251 | 3 | HDL | 0.0200 | 0.00350 | C | T | C | T | 0.028059 | 0.032916 | -0.013638 | 0.053827 | -0.020463 | 0.031151 |
| rs17404153 | 3 | HDL | 0.0280 | 0.00500 | T | G | T | G | -0.030940 | 0.048472 | -0.026512 | 0.079012 | 0.008236 | 0.046351 |
| rs13107325 | 4 | HDL | 0.0710 | 0.00780 | T | C | T | C | 0.054797 | 0.061957 | -0.050524 | 0.096669 | 0.003967 | 0.057305 |
| rs10019888 | 4 | HDL | 0.0270 | 0.00460 | G | A | G | A | -0.071319 | 0.042309 | -0.054147 | 0.069246 | 0.074304 | 0.041806 |
| rs3822072 | 4 | HDL | 0.0250 | 0.00340 | A | G | A | G | 0.003532 | 0.032278 | -0.027237 | 0.052442 | -0.002804 | 0.030356 |
| rs2602836 | 4 | HDL | 0.0190 | 0.00340 | G | A | G | A | 0.041882 | 0.032431 | 0.001614 | 0.052938 | 0.063732 | 0.030477 |
| rs6450176 | 5 | HDL | 0.0250 | 0.00390 | A | G | A | G | 0.014960 | 0.037072 | -0.128253 | 0.058336 | -0.017413 | 0.034584 |
| rs605066 | 6 | HDL | 0.0280 | 0.00490 | C | T | C | T | -0.044265 | 0.032508 | -0.035078 | 0.052936 | -0.065342 | 0.030526 |
| rs1936800 | 6 | HDL | 0.0200 | 0.00340 | T | C | T | C | 0.000586 | 0.032145 | 0.051610 | 0.052269 | -0.016909 | 0.030264 |
| rs998584 | 6 | HDL | 0.0260 | 0.00380 | A | C | A | C | -0.019493 | 0.032189 | 0.014023 | 0.052384 | -0.026654 | 0.030273 |
| rs4731702 | 7 | HDL | 0.0290 | 0.00340 | C | T | C | T | 0.050493 | 0.032095 | 0.098154 | 0.052274 | -0.034872 | 0.030208 |
| rs17145738 | 7 | HDL | 0.0410 | 0.00530 | C | T | C | T | 0.023591 | 0.048340 | -0.034379 | 0.080518 | 0.003995 | 0.045891 |
| rs702485 | 7 | HDL | 0.0240 | 0.00340 | A | G | A | G | -0.004710 | 0.032204 | 0.022942 | 0.052371 | 0.032678 | 0.030262 |
| rs4142995 | 7 | HDL | 0.0260 | 0.00370 | T | G | T | G | -0.012829 | 0.032772 | 0.039818 | 0.053647 | -0.031076 | 0.030789 |
| rs4917014 | 7 | HDL | 0.0220 | 0.00360 | T | G | T | G | -0.016021 | 0.034589 | -0.049643 | 0.056625 | -0.013805 | 0.032512 |
| rs17173637 | 7 | HDL | 0.0360 | 0.00570 | C | T | C | T | 0.008450 | 0.058297 | -0.164817 | 0.088209 | -0.038160 | 0.053590 |
| rs9987289 | 8 | HDL | 0.0820 | 0.00620 | A | G | A | G | 0.083735 | 0.057667 | -0.011751 | 0.090261 | -0.029556 | 0.051775 |
| rs2293889 | 8 | HDL | 0.0310 | 0.00350 | T | G | T | G | 0.068517 | 0.032576 | 0.055439 | 0.052998 | 0.009009 | 0.030542 |
| rs12678919 | 8 | HDL | 0.1550 | 0.00570 | A | G | A | G | -0.170809 | 0.057997 | 0.156452 | 0.082754 | 0.000477 | 0.050856 |
| rs2954029 | 8 | HDL | 0.0400 | 0.00340 | A | T | A | T | -0.091425 | 0.032397 | -0.051907 | 0.052603 | -0.047978 | 0.030413 |
| rs581080 | 9 | HDL | 0.0420 | 0.00450 | G | C | G | C | -0.010726 | 0.041577 | 0.083146 | 0.069754 | -0.044438 | 0.038713 |
| rs1883025 | 9 | HDL | 0.0700 | 0.00410 | T | C | T | C | -0.021103 | 0.036637 | -0.035850 | 0.059421 | 0.013494 | 0.034782 |
| rs970548 | 10 | HDL | 0.0260 | 0.00390 | A | C | A | C | 0.013407 | 0.037141 | 0.048269 | 0.059881 | -0.015589 | 0.035167 |
| rs2923084 | 11 | HDL | 0.0260 | 0.00450 | G | A | G | A | 0.006273 | 0.042021 | 0.057674 | 0.069530 | -0.014495 | 0.039256 |
| rs3136441 | 11 | HDL | 0.0540 | 0.00470 | T | C | T | C | 0.027138 | 0.046119 | 0.014246 | 0.075383 | 0.023821 | 0.043428 |
| rs7941030 | 11 | HDL | 0.0270 | 0.00300 | T | C | T | C | 0.025231 | 0.032863 | -0.013300 | 0.053719 | -0.018060 | 0.031081 |
| rs174546 | 11 | HDL | 0.0390 | 0.00350 | T | C | T | C | 0.062850 | 0.034017 | -0.051586 | 0.054439 | -0.026177 | 0.031589 |
| rs964184 | 11 | HDL | 0.1060 | 0.00710 | G | C | G | C | -0.055073 | 0.046349 | -0.058921 | 0.075216 | -0.064093 | 0.043445 |
| rs11246602 | 11 | HDL | 0.0340 | 0.00520 | T | C | T | C | -0.084282 | 0.048676 | 0.132328 | 0.073290 | -0.031340 | 0.044893 |
| rs12801636 | 11 | HDL | 0.0240 | 0.00420 | G | A | G | A | -0.076415 | 0.039212 | -0.016482 | 0.062761 | 0.000083 | 0.036142 |
| rs499974 | 11 | HDL | 0.0260 | 0.00440 | A | C | A | C | 0.018662 | 0.044480 | -0.024775 | 0.071326 | -0.106442 | 0.040123 |
| rs7134375 | 12 | HDL | 0.0210 | 0.00350 | C | A | C | A | -0.039298 | 0.032453 | 0.005611 | 0.052638 | -0.054489 | 0.030586 |
| rs7134594 | 12 | HDL | 0.0350 | 0.00480 | C | T | C | T | -0.009033 | 0.032068 | 0.025264 | 0.052258 | 0.035193 | 0.030213 |
| rs4759375 | 12 | HDL | 0.0560 | 0.01020 | C | T | C | T | -0.079598 | 0.060913 | -0.001653 | 0.096234 | -0.002077 | 0.055699 |
| rs4765127 | 12 | HDL | 0.0320 | 0.00500 | G | T | G | T | -0.011840 | 0.034046 | -0.005288 | 0.055318 | -0.054394 | 0.032262 |
| rs838880 | 12 | HDL | 0.0480 | 0.00390 | T | C | T | C | 0.006504 | 0.034646 | 0.070050 | 0.055716 | 0.008383 | 0.032584 |
| rs11613352 | 12 | HDL | 0.0280 | 0.00400 | C | T | C | T | 0.051041 | 0.037023 | 0.133805 | 0.059045 | -0.067215 | 0.035858 |
| rs4983559 | 14 | HDL | 0.0200 | 0.00360 | A | G | A | G | -0.030090 | 0.033120 | -0.064190 | 0.054136 | -0.023264 | 0.031134 |
| rs1532085 | 15 | HDL | 0.1070 | 0.00350 | G | A | G | A | 0.047061 | 0.032791 | 0.032648 | 0.053436 | -0.016239 | 0.031060 |
| rs2652834 | 15 | HDL | 0.0280 | 0.00430 | A | G | A | G | 0.020054 | 0.041493 | -0.082488 | 0.065398 | 0.048987 | 0.039371 |
| rs3764261 | 16 | HDL | 0.2410 | 0.00390 | C | A | C | A | -0.000413 | 0.034328 | -0.076582 | 0.056629 | -0.007196 | 0.032333 |
| rs16942887 | 16 | HDL | 0.0830 | 0.00510 | G | A | G | A | 0.088155 | 0.048702 | 0.108891 | 0.078585 | -0.086854 | 0.048894 |
| rs2925979 | 16 | HDL | 0.0350 | 0.00370 | T | C | T | C | -0.086042 | 0.034482 | 0.065901 | 0.057789 | -0.089120 | 0.032408 |
| rs1121980 | 16 | HDL | 0.0200 | 0.00340 | A | G | A | G | -0.109382 | 0.032246 | 0.092687 | 0.053262 | 0.051983 | 0.030677 |
| rs11869286 | 17 | HDL | 0.0320 | 0.00370 | G | C | G | C | -0.001036 | 0.034087 | 0.032581 | 0.055765 | -0.052983 | 0.031790 |
| rs4148008 | 17 | HDL | 0.0280 | 0.00380 | G | C | G | C | -0.048857 | 0.034183 | -0.002768 | 0.056063 | -0.017644 | 0.032346 |
| rs4129767 | 17 | HDL | 0.0240 | 0.00340 | G | A | G | A | 0.096604 | 0.032082 | 0.030761 | 0.052201 | -0.010493 | 0.030188 |
| rs7241918 | 18 | HDL | 0.0900 | 0.00640 | G | T | G | T | -0.027039 | 0.041803 | -0.133822 | 0.065980 | -0.019444 | 0.039499 |
| rs12967135 | 18 | HDL | 0.0260 | 0.00450 | A | G | A | G | -0.040927 | 0.037609 | 0.000060 | 0.061808 | 0.033930 | 0.036030 |
| rs7255436 | 19 | HDL | 0.0320 | 0.00530 | C | A | C | A | -0.055935 | 0.032138 | -0.026319 | 0.052303 | 0.039981 | 0.030277 |
| rs737337 | 19 | HDL | 0.0560 | 0.00610 | C | T | C | T | -0.012109 | 0.060321 | -0.101128 | 0.094426 | 0.013120 | 0.057233 |
| rs386000 | 19 | HDL | 0.0480 | 0.00470 | G | C | G | C | -0.005883 | 0.038145 | -0.119965 | 0.064181 | -0.087025 | 0.036766 |
| rs4420638 | 19 | HDL | 0.0670 | 0.00680 | G | A | G | A | -0.112093 | 0.039680 | -0.028141 | 0.066155 | 0.011768 | 0.038719 |
| rs17695224 | 19 | HDL | 0.0290 | 0.00390 | A | G | A | G | 0.046730 | 0.036736 | 0.086275 | 0.060337 | 0.044551 | 0.034528 |
| rs731839 | 19 | HDL | 0.0220 | 0.00370 | G | A | G | A | -0.011383 | 0.034005 | -0.031631 | 0.055162 | 0.092376 | 0.032579 |
| rs1800961 | 20 | HDL | 0.1270 | 0.00990 | T | C | T | C | 0.107022 | 0.096680 | -0.040720 | 0.146938 | 0.267857 | 0.098174 |
| rs6065906 | 20 | HDL | 0.0590 | 0.00440 | C | T | C | T | -0.059018 | 0.040422 | -0.027710 | 0.066451 | -0.048180 | 0.038194 |
| rs181362 | 22 | HDL | 0.0380 | 0.00300 | T | C | T | C | 0.042486 | 0.041410 | 0.050407 | 0.067443 | 0.035899 | 0.038783 |
| Chr: chromosome; beta: regression coefficient; se: standard error; AS: aortic stenosis; AR: aortic regurgitation; MR: mitral regurgitation  Summary statistics related to SNP-HDL extracted from Global Lipids Genetics Consortium 2013 [Discovery and refinement of loci associated with lipid levels; Nature Genetics volume 45, pages 1274–1283 (2013)] and corresponding SNP-outcomes estimations calculated using individual participants data of the UK Biobank. | | | | | | | | | | | | | | |

| Dataset 2. Harmonized dataset of two-sample Mendelian randomization for the effect of LDL cholesterol on valvular heart disease. | | | | | | | | | | | | | | |
| --- | --- | --- | --- | --- | --- | --- | --- | --- | --- | --- | --- | --- | --- | --- |
| SNP | Chr | Trait | beta_LDL | se_LDL | effect_allele_LDL | other_allele_LDL | effect_allele_outcome | other_allele_outcome | beta_AS | se_AS | beta_AR | se_AR | beta_MR | se_MR |
| rs2479409 | 1 | LDL | 0.0640 | 0.0041 | A | G | A | G | 0.0749138 | 0.0332969 | 0.0023209 | 0.0547479 | -0.0031194 | 0.0316757 |
| rs629301 | 1 | LDL | 0.1670 | 0.0049 | G | T | G | T | 0.1143679 | 0.0398164 | -0.0957064 | 0.0611978 | 0.0533734 | 0.0368574 |
| rs12027135 | 1 | LDL | 0.0300 | 0.0038 | A | T | A | T | -0.0178936 | 0.0322271 | -0.0887200 | 0.0522922 | 0.0349276 | 0.0304218 |
| rs2642442 | 1 | LDL | 0.0360 | 0.0054 | C | T | C | T | 0.0460890 | 0.0347250 | 0.0380681 | 0.0564391 | 0.0433816 | 0.0326722 |
| rs514230 | 1 | LDL | 0.0360 | 0.0054 | A | T | A | T | 0.0328277 | 0.0321736 | 0.0140526 | 0.0523436 | 0.0303663 | 0.0302731 |
| rs2131925 | 1 | LDL | 0.0490 | 0.0039 | G | T | G | T | 0.0086050 | 0.0336462 | -0.0752229 | 0.0541417 | 0.0372909 | 0.0317884 |
| rs12748152 | 1 | LDL | 0.0500 | 0.0066 | C | T | C | T | 0.0425179 | 0.0579198 | 0.0749690 | 0.0930640 | -0.0919721 | 0.0576519 |
| rs267733 | 1 | LDL | 0.0330 | 0.0053 | G | A | G | A | -0.0026665 | 0.0435230 | -0.0208611 | 0.0704642 | -0.0255665 | 0.0406607 |
| rs1367117 | 2 | LDL | 0.1190 | 0.0040 | G | A | G | A | 0.0034413 | 0.0339198 | -0.0471061 | 0.0557013 | -0.0553945 | 0.0322294 |
| rs4299376 | 2 | LDL | 0.0810 | 0.0045 | T | G | T | G | 0.0671408 | 0.0339532 | 0.0708897 | 0.0551970 | 0.0404926 | 0.0320850 |
| rs2710642 | 2 | LDL | 0.0240 | 0.0038 | G | A | G | A | 0.0560399 | 0.0345988 | -0.0634608 | 0.0551430 | -0.0447331 | 0.0319859 |
| rs10490626 | 2 | LDL | 0.0510 | 0.0069 | A | G | A | G | 0.0369200 | 0.0584737 | -0.0418521 | 0.0922803 | 0.1001613 | 0.0566412 |
| rs2030746 | 2 | LDL | 0.0210 | 0.0038 | C | T | C | T | -0.0075897 | 0.0325854 | -0.0005021 | 0.0529944 | 0.0056309 | 0.0306289 |
| rs1250229 | 2 | LDL | 0.0240 | 0.0042 | T | C | T | C | 0.0011680 | 0.0367133 | 0.0487172 | 0.0603985 | 0.0224412 | 0.0347018 |
| rs11563251 | 2 | LDL | 0.0340 | 0.0062 | C | T | C | T | -0.0471401 | 0.0519363 | 0.0888371 | 0.0801908 | -0.0674900 | 0.0492757 |
| rs7640978 | 3 | LDL | 0.0390 | 0.0069 | T | C | T | C | 0.1064456 | 0.0589982 | 0.2085827 | 0.1000780 | -0.0390185 | 0.0522196 |
| rs17404153 | 3 | LDL | 0.0340 | 0.0054 | T | G | T | G | -0.0309395 | 0.0484720 | -0.0265123 | 0.0790120 | 0.0082357 | 0.0463511 |
| rs6831256 | 4 | LDL | 0.0220 | 0.0038 | A | G | A | G | 0.0816555 | 0.0323296 | -0.0400546 | 0.0530595 | -0.0410507 | 0.0306888 |
| rs12916 | 5 | LDL | 0.0730 | 0.0038 | T | C | T | C | -0.0202751 | 0.0327849 | 0.0276861 | 0.0531285 | -0.0350463 | 0.0309068 |
| rs6882076 | 5 | LDL | 0.0460 | 0.0038 | T | C | T | C | -0.0160475 | 0.0332371 | -0.0212157 | 0.0540455 | 0.0392222 | 0.0315006 |
| rs4530754 | 5 | LDL | 0.0280 | 0.0036 | G | A | G | A | -0.0835971 | 0.0321103 | -0.0225508 | 0.0523505 | -0.0239265 | 0.0302651 |
| rs3757354 | 6 | LDL | 0.0380 | 0.0044 | T | C | T | C | -0.0141311 | 0.0397017 | 0.0258521 | 0.0654095 | 0.0336806 | 0.0378877 |
| rs1800562 | 6 | LDL | 0.0620 | 0.0080 | A | G | A | G | 0.0179673 | 0.0599748 | -0.0416038 | 0.0956237 | 0.0048835 | 0.0564833 |
| rs1564348 | 6 | LDL | 0.0480 | 0.0050 | T | C | T | C | -0.0148107 | 0.0429997 | 0.0098862 | 0.0694050 | 0.0526758 | 0.0395997 |
| rs3177928 | 6 | LDL | 0.0450 | 0.0052 | G | A | G | A | 0.0435586 | 0.0433028 | -0.0283693 | 0.0722332 | -0.0677734 | 0.0422768 |
| rs9488822 | 6 | LDL | 0.0310 | 0.0054 | A | T | A | T | 0.0439593 | 0.0332098 | 0.0912278 | 0.0537740 | -0.0126862 | 0.0314911 |
| rs12670798 | 7 | LDL | 0.0340 | 0.0043 | T | C | T | C | -0.0046921 | 0.0372796 | 0.0055646 | 0.0605087 | 0.0853605 | 0.0343005 |
| rs2072183 | 7 | LDL | 0.0390 | 0.0047 | G | C | G | C | 0.0412534 | 0.0381926 | -0.1471644 | 0.0655563 | -0.0208570 | 0.0365063 |
| rs4722551 | 7 | LDL | 0.0390 | 0.0049 | T | C | T | C | -0.0115109 | 0.0439906 | 0.0598453 | 0.0698830 | 0.0593951 | 0.0404408 |
| rs9987289 | 8 | LDL | 0.0710 | 0.0066 | A | G | A | G | 0.0837345 | 0.0576668 | -0.0117505 | 0.0902612 | -0.0295563 | 0.0517746 |
| rs11136341 | 8 | LDL | 0.0450 | 0.0062 | A | G | A | G | -0.0394789 | 0.0331962 | -0.0269492 | 0.0539531 | 0.0014015 | 0.0310836 |
| rs2081687 | 8 | LDL | 0.0310 | 0.0054 | C | T | T | C | -0.0117480 | 0.0338672 | 0.1027554 | 0.0562275 | 0.0045972 | 0.0319590 |
| rs2954029 | 8 | LDL | 0.0560 | 0.0036 | T | A | T | A | 0.0914245 | 0.0323968 | 0.0519070 | 0.0526029 | 0.0479783 | 0.0304128 |
| rs10102164 | 8 | LDL | 0.0320 | 0.0045 | G | A | G | A | 0.0309185 | 0.0387611 | 0.0052967 | 0.0636018 | 0.0228618 | 0.0365865 |
| rs3780181 | 9 | LDL | 0.0440 | 0.0074 | G | A | G | A | 0.0754723 | 0.0665353 | 0.2866688 | 0.1189066 | 0.0373716 | 0.0617450 |
| rs2255141 | 10 | LDL | 0.0300 | 0.0040 | G | A | G | A | 0.0565247 | 0.0355736 | 0.0276712 | 0.0582100 | -0.0461692 | 0.0342080 |
| rs11220462 | 11 | LDL | 0.0590 | 0.0059 | G | A | G | A | 0.0369152 | 0.0467043 | -0.1566662 | 0.0816584 | -0.0082563 | 0.0446537 |
| rs174546 | 11 | LDL | 0.0510 | 0.0038 | T | C | T | C | 0.0628498 | 0.0340173 | -0.0515857 | 0.0544388 | -0.0261773 | 0.0315889 |
| rs964184 | 11 | LDL | 0.0860 | 0.0078 | C | G | C | G | 0.0550729 | 0.0463493 | 0.0589212 | 0.0752156 | 0.0640925 | 0.0434447 |
| rs11065987 | 12 | LDL | 0.0270 | 0.0038 | G | A | G | A | -0.1082952 | 0.0322905 | -0.0905446 | 0.0526328 | 0.0381118 | 0.0307274 |
| rs1169288 | 12 | LDL | 0.0380 | 0.0040 | A | C | A | C | 0.0726115 | 0.0342865 | -0.0150736 | 0.0566864 | 0.0662756 | 0.0323126 |
| rs4942486 | 13 | LDL | 0.0240 | 0.0037 | C | T | C | T | 0.0448192 | 0.0321077 | 0.1560656 | 0.0522347 | 0.0441438 | 0.0302111 |
| rs8017377 | 14 | LDL | 0.0300 | 0.0038 | G | A | G | A | -0.0117953 | 0.0321215 | -0.0374703 | 0.0523123 | -0.0393226 | 0.0302527 |
| rs3764261 | 16 | LDL | 0.0530 | 0.0042 | A | C | A | C | 0.0004131 | 0.0343283 | 0.0765823 | 0.0566289 | 0.0071959 | 0.0323327 |
| rs2000999 | 16 | LDL | 0.0650 | 0.0046 | G | A | G | A | -0.0405448 | 0.0415539 | -0.0147875 | 0.0670369 | 0.0049152 | 0.0385204 |
| rs7206971 | 17 | LDL | 0.0290 | 0.0055 | G | A | G | A | 0.0153276 | 0.0321587 | -0.0495485 | 0.0523587 | -0.0030414 | 0.0302576 |
| rs1801689 | 17 | LDL | 0.1030 | 0.0139 | A | C | A | C | -0.0284573 | 0.0947999 | -0.3141298 | 0.1763919 | 0.0019153 | 0.0881871 |
| rs314253 | 17 | LDL | 0.0240 | 0.0038 | C | T | C | T | 0.0307763 | 0.0337526 | -0.0121528 | 0.0545920 | 0.0416461 | 0.0318245 |
| rs6511720 | 19 | LDL | 0.2210 | 0.0061 | T | G | T | G | 0.0995318 | 0.0516323 | 0.0360006 | 0.0818851 | 0.0277269 | 0.0471908 |
| rs4420638 | 19 | LDL | 0.2250 | 0.0077 | A | G | A | G | 0.1120934 | 0.0396799 | 0.0281410 | 0.0661551 | -0.0117676 | 0.0387186 |
| rs10401969 | 19 | LDL | 0.1180 | 0.0072 | C | T | C | T | 0.0648915 | 0.0621171 | 0.0643654 | 0.1010481 | 0.0900998 | 0.0589700 |
| rs6029526 | 20 | LDL | 0.0440 | 0.0052 | T | A | T | A | 0.0641022 | 0.0321439 | -0.0174725 | 0.0523746 | -0.0075940 | 0.0302712 |
| rs2902940 | 20 | LDL | 0.0270 | 0.0041 | G | A | G | A | -0.0519083 | 0.0340067 | -0.0902730 | 0.0550024 | -0.0418589 | 0.0321007 |
| rs364585 | 20 | LDL | 0.0250 | 0.0038 | A | G | A | G | 0.0806795 | 0.0331779 | 0.0561092 | 0.0538396 | -0.0309432 | 0.0308361 |
| rs2328223 | 20 | LDL | 0.0300 | 0.0050 | A | C | A | C | 0.0117321 | 0.0411202 | 0.0852241 | 0.0653211 | 0.0265526 | 0.0384492 |
| rs5763662 | 22 | LDL | 0.0770 | 0.0121 | C | T | C | T | -0.0013264 | 0.1089948 | -0.0553199 | 0.1818762 | -0.1003821 | 0.1075399 |
| rs4253772 | 22 | LDL | 0.0310 | 0.0060 | C | T | C | T | 0.0197478 | 0.0506398 | -0.0450240 | 0.0845322 | -0.0589464 | 0.0491569 |
| Chr: chromosome; beta: regression coefficient; se: standard error; AS: aortic stenosis; AR: aortic regurgitation; MR: mitral regurgitation  Summary statistics related to SNP-LDL extracted from Global Lipids Genetics Consortium 2013 [Discovery and refinement of loci associated with lipid levels; Nature Genetics volume 45, pages 1274–1283 (2013)] and corresponding SNP-outcomes estimations calculated using individual participants data of the UK Biobank. | | | | | | | | | | | | | | |

| Dataset 3. Harmonized dataset of two-sample Mendelian randomization for the effect of total cholesterol on valvular heart disease. | | | | | | | | | | | | | | |
| --- | --- | --- | --- | --- | --- | --- | --- | --- | --- | --- | --- | --- | --- | --- |
| SNP | Chr | Trait | beta_TC | se_TC | effect_allele_TC | other_allele_TC | effect_allele_outcome | other_allele_outcome | beta_AS | se_AS | beta_AR | se_AR | beta_MR | se_MR |
| rs2479409 | 1 | TC | 0.0540 | 0.0040 | A | G | A | G | 0.0749138 | 0.0332969 | 0.0023209 | 0.0547479 | -0.0031194 | 0.0316757 |
| rs629301 | 1 | TC | 0.1340 | 0.0047 | G | T | G | T | 0.1143679 | 0.0398164 | -0.0957064 | 0.0611978 | 0.0533734 | 0.0368574 |
| rs12027135 | 1 | TC | 0.0270 | 0.0036 | A | T | A | T | -0.0178936 | 0.0322271 | -0.0887200 | 0.0522922 | 0.0349276 | 0.0304218 |
| rs7515577 | 1 | TC | 0.0370 | 0.0063 | C | A | C | A | 0.0023820 | 0.0395931 | 0.0509654 | 0.0653870 | -0.0003260 | 0.0371774 |
| rs2642442 | 1 | TC | 0.0350 | 0.0052 | C | T | C | T | 0.0460890 | 0.0347250 | 0.0380681 | 0.0564391 | 0.0433816 | 0.0326722 |
| rs514230 | 1 | TC | 0.0390 | 0.0053 | A | T | A | T | 0.0328277 | 0.0321736 | 0.0140526 | 0.0523436 | 0.0303663 | 0.0302731 |
| rs2131925 | 1 | TC | 0.0750 | 0.0037 | G | T | G | T | 0.0086050 | 0.0336462 | -0.0752229 | 0.0541417 | 0.0372909 | 0.0317884 |
| rs1077514 | 1 | TC | 0.0300 | 0.0052 | C | T | C | T | 0.0346319 | 0.0466204 | -0.0336730 | 0.0740719 | 0.0781111 | 0.0445198 |
| rs1367117 | 2 | TC | 0.1000 | 0.0038 | G | A | G | A | 0.0034413 | 0.0339198 | -0.0471061 | 0.0557013 | -0.0553945 | 0.0322294 |
| rs4299376 | 2 | TC | 0.0790 | 0.0043 | T | G | T | G | 0.0671408 | 0.0339532 | 0.0708897 | 0.0551970 | 0.0404926 | 0.0320850 |
| rs7570971 | 2 | TC | 0.0300 | 0.0039 | C | A | C | A | -0.0613316 | 0.0372748 | -0.1066470 | 0.0612435 | -0.0343032 | 0.0347232 |
| rs1260326 | 2 | TC | 0.0510 | 0.0036 | C | T | C | T | 0.0839378 | 0.0325478 | -0.0107029 | 0.0534679 | -0.0103630 | 0.0309069 |
| rs10490626 | 2 | TC | 0.0420 | 0.0066 | A | G | A | G | 0.0369200 | 0.0584737 | -0.0418521 | 0.0922803 | 0.1001613 | 0.0566412 |
| rs2030746 | 2 | TC | 0.0200 | 0.0037 | C | T | C | T | -0.0075897 | 0.0325854 | -0.0005021 | 0.0529944 | 0.0056309 | 0.0306289 |
| rs2287623 | 2 | TC | 0.0270 | 0.0036 | A | G | A | G | 0.0126296 | 0.0327585 | -0.0561649 | 0.0537104 | -0.0425220 | 0.0310200 |
| rs11694172 | 2 | TC | 0.0280 | 0.0041 | A | G | A | G | 0.0707688 | 0.0365913 | -0.0319269 | 0.0610029 | 0.0254173 | 0.0347954 |
| rs11563251 | 2 | TC | 0.0370 | 0.0059 | C | T | C | T | -0.0471401 | 0.0519363 | 0.0888371 | 0.0801908 | -0.0674900 | 0.0492757 |
| rs2290159 | 3 | TC | 0.0370 | 0.0061 | C | G | C | G | -0.0256353 | 0.0386093 | -0.0169401 | 0.0630253 | 0.0077889 | 0.0367107 |
| rs7640978 | 3 | TC | 0.0380 | 0.0066 | T | C | T | C | 0.1064456 | 0.0589982 | 0.2085827 | 0.1000780 | -0.0390185 | 0.0522196 |
| rs13315871 | 3 | TC | 0.0360 | 0.0061 | A | G | A | G | 0.0152585 | 0.0571195 | -0.0662826 | 0.0897955 | -0.0048138 | 0.0532769 |
| rs6831256 | 4 | TC | 0.0250 | 0.0037 | A | G | A | G | 0.0816555 | 0.0323296 | -0.0400546 | 0.0530595 | -0.0410507 | 0.0306888 |
| rs12916 | 5 | TC | 0.0680 | 0.0036 | T | C | T | C | -0.0202751 | 0.0327849 | 0.0276861 | 0.0531285 | -0.0350463 | 0.0309068 |
| rs6882076 | 5 | TC | 0.0510 | 0.0037 | T | C | T | C | -0.0160475 | 0.0332371 | -0.0212157 | 0.0540455 | 0.0392222 | 0.0315006 |
| rs4530754 | 5 | TC | 0.0230 | 0.0035 | G | A | G | A | -0.0835971 | 0.0321103 | -0.0225508 | 0.0523505 | -0.0239265 | 0.0302651 |
| rs3757354 | 6 | TC | 0.0350 | 0.0042 | T | C | T | C | -0.0141311 | 0.0397017 | 0.0258521 | 0.0654095 | 0.0336806 | 0.0378877 |
| rs1800562 | 6 | TC | 0.0560 | 0.0077 | A | G | A | G | 0.0179673 | 0.0599748 | -0.0416038 | 0.0956237 | 0.0048835 | 0.0564833 |
| rs1564348 | 6 | TC | 0.0490 | 0.0048 | T | C | T | C | -0.0148107 | 0.0429997 | 0.0098862 | 0.0694050 | 0.0526758 | 0.0395997 |
| rs3177928 | 6 | TC | 0.0480 | 0.0050 | G | A | G | A | 0.0435586 | 0.0433028 | -0.0283693 | 0.0722332 | -0.0677734 | 0.0422768 |
| rs2814982 | 6 | TC | 0.0440 | 0.0057 | T | C | T | C | 0.0197559 | 0.0534798 | 0.0137024 | 0.0867903 | -0.0625056 | 0.0486911 |
| rs9488822 | 6 | TC | 0.0340 | 0.0053 | A | T | A | T | 0.0439593 | 0.0332098 | 0.0912278 | 0.0537740 | -0.0126862 | 0.0314911 |
| rs2758886 | 6 | TC | 0.0230 | 0.0039 | G | A | G | A | -0.0254854 | 0.0352623 | 0.1007686 | 0.0559874 | -0.0169824 | 0.0331095 |
| rs9376090 | 6 | TC | 0.0250 | 0.0040 | C | T | C | T | 0.0528263 | 0.0371163 | 0.0472363 | 0.0603408 | 0.0274854 | 0.0347017 |
| rs12670798 | 7 | TC | 0.0360 | 0.0041 | T | C | T | C | -0.0046921 | 0.0372796 | 0.0055646 | 0.0605087 | 0.0853605 | 0.0343005 |
| rs2072183 | 7 | TC | 0.0360 | 0.0045 | G | C | G | C | 0.0412534 | 0.0381926 | -0.1471644 | 0.0655563 | -0.0208570 | 0.0365063 |
| rs4722551 | 7 | TC | 0.0290 | 0.0047 | T | C | T | C | -0.0115109 | 0.0439906 | 0.0598453 | 0.0698830 | 0.0593951 | 0.0404408 |
| rs1997243 | 7 | TC | 0.0330 | 0.0050 | A | G | A | G | 0.0147581 | 0.0451811 | 0.0450244 | 0.0726400 | -0.0103137 | 0.0427941 |
| rs9987289 | 8 | TC | 0.0840 | 0.0063 | A | G | A | G | 0.0837345 | 0.0576668 | -0.0117505 | 0.0902612 | -0.0295563 | 0.0517746 |
| rs11136341 | 8 | TC | 0.0380 | 0.0061 | A | G | A | G | -0.0394789 | 0.0331962 | -0.0269492 | 0.0539531 | 0.0014015 | 0.0310836 |
| rs2081687 | 8 | TC | 0.0380 | 0.0052 | C | T | C | T | 0.0117480 | 0.0338672 | -0.1027554 | 0.0562275 | -0.0045972 | 0.0319590 |
| rs1495741 | 8 | TC | 0.0320 | 0.0061 | A | G | A | G | 0.0055084 | 0.0385935 | 0.0115900 | 0.0627076 | 0.0196633 | 0.0361466 |
| rs2954029 | 8 | TC | 0.0620 | 0.0035 | T | A | T | A | 0.0914245 | 0.0323968 | 0.0519070 | 0.0526029 | 0.0479783 | 0.0304128 |
| rs10102164 | 8 | TC | 0.0300 | 0.0043 | G | A | G | A | 0.0309185 | 0.0387611 | 0.0052967 | 0.0636018 | 0.0228618 | 0.0365865 |
| rs581080 | 9 | TC | 0.0380 | 0.0047 | G | C | G | C | -0.0107263 | 0.0415770 | 0.0831461 | 0.0697535 | -0.0444382 | 0.0387132 |
| rs1883025 | 9 | TC | 0.0670 | 0.0042 | T | C | T | C | -0.0211028 | 0.0366371 | -0.0358498 | 0.0594206 | 0.0134937 | 0.0347821 |
| rs3780181 | 9 | TC | 0.0440 | 0.0071 | G | A | G | A | 0.0754723 | 0.0665353 | 0.2866688 | 0.1189066 | 0.0373716 | 0.0617450 |
| rs2255141 | 10 | TC | 0.0310 | 0.0039 | G | A | G | A | 0.0565247 | 0.0355736 | 0.0276712 | 0.0582100 | -0.0461692 | 0.0342080 |
| rs970548 | 10 | TC | 0.0250 | 0.0040 | A | C | A | C | 0.0134074 | 0.0371409 | 0.0482687 | 0.0598805 | -0.0155888 | 0.0351667 |
| rs10904908 | 10 | TC | 0.0250 | 0.0036 | A | G | A | G | 0.0362530 | 0.0324480 | 0.0019681 | 0.0529362 | 0.0702117 | 0.0304505 |
| rs11220462 | 11 | TC | 0.0470 | 0.0058 | G | A | G | A | 0.0369152 | 0.0467043 | -0.1566662 | 0.0816584 | -0.0082563 | 0.0446537 |
| rs10128711 | 11 | TC | 0.0310 | 0.0043 | T | C | T | C | 0.0164658 | 0.0366788 | 0.0665212 | 0.0604444 | 0.0080068 | 0.0344606 |
| rs7941030 | 11 | TC | 0.0280 | 0.0040 | T | C | T | C | 0.0252311 | 0.0328633 | -0.0133003 | 0.0537187 | -0.0180599 | 0.0310811 |
| rs174546 | 11 | TC | 0.0480 | 0.0036 | T | C | T | C | 0.0628498 | 0.0340173 | -0.0515857 | 0.0544388 | -0.0261773 | 0.0315889 |
| rs964184 | 11 | TC | 0.1210 | 0.0076 | C | G | C | G | 0.0550729 | 0.0463493 | 0.0589212 | 0.0752156 | 0.0640925 | 0.0434447 |
| rs11603023 | 11 | TC | 0.0220 | 0.0036 | C | T | C | T | -0.0050541 | 0.0323399 | 0.0128120 | 0.0525617 | -0.0349262 | 0.0305015 |
| rs11065987 | 12 | TC | 0.0310 | 0.0036 | G | A | G | A | -0.1082952 | 0.0322905 | -0.0905446 | 0.0526328 | 0.0381118 | 0.0307274 |
| rs1169288 | 12 | TC | 0.0320 | 0.0039 | A | C | A | C | 0.0726115 | 0.0342865 | -0.0150736 | 0.0566864 | 0.0662756 | 0.0323126 |
| rs4883201 | 12 | TC | 0.0350 | 0.0056 | G | A | G | A | -0.0576609 | 0.0517241 | -0.0941868 | 0.0829953 | 0.0310433 | 0.0503938 |
| rs1532085 | 15 | TC | 0.0540 | 0.0036 | G | A | G | A | 0.0470610 | 0.0327911 | 0.0326482 | 0.0534356 | -0.0162393 | 0.0310603 |
| rs3764261 | 16 | TC | 0.0500 | 0.0040 | C | A | C | A | -0.0004131 | 0.0343283 | -0.0765823 | 0.0566289 | -0.0071959 | 0.0323327 |
| rs2000999 | 16 | TC | 0.0620 | 0.0044 | G | A | G | A | -0.0405448 | 0.0415539 | -0.0147875 | 0.0670369 | 0.0049152 | 0.0385204 |
| rs7206971 | 17 | TC | 0.0300 | 0.0054 | G | A | G | A | 0.0153276 | 0.0321587 | -0.0495485 | 0.0523587 | -0.0030414 | 0.0302576 |
| rs314253 | 17 | TC | 0.0230 | 0.0037 | C | T | C | T | 0.0307763 | 0.0337526 | -0.0121528 | 0.0545920 | 0.0416461 | 0.0318245 |
| rs7241918 | 18 | TC | 0.0580 | 0.0068 | G | T | G | T | -0.0270393 | 0.0418030 | -0.1338215 | 0.0659798 | -0.0194436 | 0.0394986 |
| rs6511720 | 19 | TC | 0.1850 | 0.0059 | T | G | T | G | 0.0995318 | 0.0516323 | 0.0360006 | 0.0818851 | 0.0277269 | 0.0471908 |
| rs4420638 | 19 | TC | 0.1970 | 0.0073 | A | G | A | G | 0.1120934 | 0.0396799 | 0.0281410 | 0.0661551 | -0.0117676 | 0.0387186 |
| rs10401969 | 19 | TC | 0.1370 | 0.0070 | C | T | C | T | 0.0648915 | 0.0621171 | 0.0643654 | 0.1010481 | 0.0900998 | 0.0589700 |
| rs492602 | 19 | TC | 0.0310 | 0.0037 | A | G | A | G | 0.0307764 | 0.0322391 | -0.0256904 | 0.0524127 | -0.0245807 | 0.0303045 |
| rs1800961 | 20 | TC | 0.1060 | 0.0101 | T | C | T | C | 0.1070224 | 0.0966800 | -0.0407195 | 0.1469379 | 0.2678573 | 0.0981737 |
| rs6029526 | 20 | TC | 0.0400 | 0.0051 | T | A | T | A | 0.0641022 | 0.0321439 | -0.0174725 | 0.0523746 | -0.0075940 | 0.0302712 |
| rs2277862 | 20 | TC | 0.0350 | 0.0052 | T | C | T | C | 0.0078832 | 0.0467529 | -0.0201354 | 0.0752315 | 0.0112235 | 0.0440113 |
| rs2902940 | 20 | TC | 0.0240 | 0.0039 | G | A | G | A | -0.0519083 | 0.0340067 | -0.0902730 | 0.0550024 | -0.0418589 | 0.0321007 |
| rs138777 | 22 | TC | 0.0210 | 0.0037 | G | A | G | A | -0.0054413 | 0.0338211 | 0.0114260 | 0.0548672 | -0.0345415 | 0.0319534 |
| rs4253772 | 22 | TC | 0.0320 | 0.0058 | C | T | C | T | 0.0197478 | 0.0506398 | -0.0450240 | 0.0845322 | -0.0589464 | 0.0491569 |
| Chr: chromosome; TC: total cholesterol; beta: regression coefficient; se: standard error; AS: aortic stenosis; AR: aortic regurgitation; MR: mitral regurgitation  Summary statistics related to SNP-TC extracted from Global Lipids Genetics Consortium 2013 [Discovery and refinement of loci associated with lipid levels; Nature Genetics volume 45, pages 1274–1283 (2013)] and corresponding SNP-outcomes estimations calculated using individual participants data of the UK Biobank. | | | | | | | | | | | | | | |

| Dataset 4. Harmonized dataset of two-sample Mendelian randomization for the effect of triglyceride on valvular heart disease. | | | | | | | | | | | | | | |
| --- | --- | --- | --- | --- | --- | --- | --- | --- | --- | --- | --- | --- | --- | --- |
| SNP | Chr | Trait | beta_TG | se_TG | effect_allele_TG | other_allele_TG | effect_allele_outcome | other_allele_outcome | beta_AS | se_AS | beta_AR | se_AR | beta_MR | se_MR |
| rs4846914 | 1 | TG | 0.0400 | 0.0034 | A | G | A | G | 0.0447351 | 0.0447351 | 0.0516823 | 0.0531774 | 0.0137849 | 0.0308597 |
| rs2131925 | 1 | TG | 0.0660 | 0.0035 | G | T | G | T | 0.0086050 | 0.0336462 | -0.0752229 | 0.0541417 | 0.0372909 | 0.0317884 |
| rs12748152 | 1 | TG | 0.0370 | 0.0059 | C | T | C | T | 0.0425179 | 0.0579198 | 0.0749690 | 0.0930640 | -0.0919721 | 0.0576519 |
| rs2972146 | 2 | TG | 0.0280 | 0.0034 | G | T | G | T | 0.0147604 | 0.0336679 | 0.0123946 | 0.0547736 | 0.0254055 | 0.0317259 |
| rs1260326 | 2 | TG | 0.1150 | 0.0034 | C | T | C | T | 0.0839378 | 0.0325478 | -0.0107029 | 0.0534679 | -0.0103630 | 0.0309069 |
| rs645040 | 3 | TG | 0.0290 | 0.0040 | G | T | G | T | 0.0223549 | 0.0384786 | -0.0382268 | 0.0616206 | 0.0457375 | 0.0364604 |
| rs442177 | 4 | TG | 0.0310 | 0.0033 | G | T | G | T | 0.0154556 | 0.0327227 | -0.0963661 | 0.0527813 | 0.0529646 | 0.0309061 |
| rs6831256 | 4 | TG | 0.0260 | 0.0035 | A | G | A | G | 0.0816555 | 0.0323296 | -0.0400546 | 0.0530595 | -0.0410507 | 0.0306888 |
| rs6882076 | 5 | TG | 0.0290 | 0.0035 | T | C | T | C | -0.0160475 | 0.0332371 | -0.0212157 | 0.0540455 | 0.0392222 | 0.0315006 |
| rs9686661 | 5 | TG | 0.0380 | 0.0044 | C | T | C | T | 0.0127632 | 0.0398622 | -0.0060210 | 0.0652075 | 0.0625408 | 0.0369694 |
| rs1936800 | 6 | TG | 0.0200 | 0.0033 | C | T | C | T | -0.0005864 | 0.0321453 | -0.0516101 | 0.0522692 | 0.0169094 | 0.0302639 |
| rs998584 | 6 | TG | 0.0290 | 0.0037 | C | A | C | A | 0.0194933 | 0.0321894 | -0.0140230 | 0.0523843 | 0.0266544 | 0.0302734 |
| rs13238203 | 7 | TG | 0.0590 | 0.0140 | T | C | T | C | -0.2847171 | 0.0839390 | 0.0204100 | 0.1565711 | -0.0274579 | 0.0885417 |
| rs17145738 | 7 | TG | 0.1150 | 0.0053 | T | C | T | C | -0.0235905 | 0.0483402 | 0.0343793 | 0.0805177 | -0.0039954 | 0.0458912 |
| rs4722551 | 7 | TG | 0.0230 | 0.0044 | T | C | T | C | -0.0115109 | 0.0439906 | 0.0598453 | 0.0698830 | 0.0593951 | 0.0404408 |
| rs38855 | 7 | TG | 0.0190 | 0.0033 | G | A | G | A | 0.0557363 | 0.0323280 | 0.0296415 | 0.0525809 | 0.0219370 | 0.0303880 |
| rs11776767 | 8 | TG | 0.0220 | 0.0035 | G | C | G | C | -0.0641589 | 0.0334646 | -0.1717870 | 0.0553784 | -0.0496677 | 0.0314330 |
| rs1495741 | 8 | TG | 0.0400 | 0.0056 | A | G | A | G | 0.0055084 | 0.0385935 | 0.0115900 | 0.0627076 | 0.0196633 | 0.0361466 |
| rs12678919 | 8 | TG | 0.1700 | 0.0056 | G | A | G | A | 0.1708087 | 0.0579967 | -0.1564522 | 0.0827537 | -0.0004770 | 0.0508555 |
| rs2954029 | 8 | TG | 0.0760 | 0.0033 | T | A | T | A | 0.0914245 | 0.0323968 | 0.0519070 | 0.0526029 | 0.0479783 | 0.0304128 |
| rs10761731 | 10 | TG | 0.0310 | 0.0047 | T | A | T | A | 0.0159749 | 0.0326532 | 0.1010395 | 0.0535844 | -0.0247330 | 0.0306187 |
| rs2068888 | 10 | TG | 0.0240 | 0.0034 | A | G | A | G | 0.0281992 | 0.0322917 | -0.0332279 | 0.0523815 | 0.0197194 | 0.0303696 |
| rs1832007 | 10 | TG | 0.0330 | 0.0047 | G | A | G | A | 0.0890279 | 0.0457587 | -0.0496509 | 0.0710761 | -0.0101752 | 0.0416311 |
| rs174546 | 11 | TG | 0.0450 | 0.0034 | C | T | C | T | -0.0628498 | 0.0340173 | 0.0515857 | 0.0544388 | 0.0261773 | 0.0315889 |
| rs964184 | 11 | TG | 0.2340 | 0.0069 | C | G | C | G | 0.0550729 | 0.0463493 | 0.0589212 | 0.0752156 | 0.0640925 | 0.0434447 |
| rs4765127 | 12 | TG | 0.0290 | 0.0049 | T | G | T | G | 0.0118395 | 0.0340461 | 0.0052875 | 0.0553178 | 0.0543939 | 0.0322620 |
| rs11613352 | 12 | TG | 0.0280 | 0.0039 | T | C | T | C | -0.0510412 | 0.0370227 | -0.1338053 | 0.0590453 | 0.0672153 | 0.0358579 |
| rs1532085 | 15 | TG | 0.0310 | 0.0034 | G | A | G | A | 0.0470610 | 0.0327911 | 0.0326482 | 0.0534356 | -0.0162393 | 0.0310603 |
| rs2412710 | 15 | TG | 0.0990 | 0.0132 | G | A | G | A | -0.2228282 | 0.1350883 | 0.2622276 | 0.1739127 | 0.0782147 | 0.1100330 |
| rs2929282 | 15 | TG | 0.0720 | 0.0121 | A | T | A | T | -0.0770343 | 0.0811868 | 0.1081161 | 0.1212429 | 0.1677959 | 0.0684319 |
| rs3764261 | 16 | TG | 0.0400 | 0.0038 | A | C | A | C | 0.0004131 | 0.0343283 | 0.0765823 | 0.0566289 | 0.0071959 | 0.0323327 |
| rs11649653 | 16 | TG | 0.0270 | 0.0050 | G | C | G | C | 0.0668145 | 0.0332649 | -0.0029352 | 0.0536499 | 0.0035563 | 0.0310381 |
| rs1121980 | 16 | TG | 0.0210 | 0.0034 | G | A | G | A | 0.1093818 | 0.0322460 | -0.0926873 | 0.0532620 | -0.0519833 | 0.0306774 |
| rs3198697 | 16 | TG | 0.0200 | 0.0034 | T | C | T | C | -0.0380587 | 0.0325525 | 0.0155566 | 0.0532366 | 0.0275548 | 0.0308072 |
| rs8077889 | 17 | TG | 0.0250 | 0.0042 | A | C | A | C | -0.0327131 | 0.0394962 | 0.0689852 | 0.0624532 | 0.0370326 | 0.0364501 |
| rs10401969 | 19 | TG | 0.1210 | 0.0065 | C | T | C | T | 0.0648915 | 0.0621171 | 0.0643654 | 0.1010481 | 0.0900998 | 0.0589700 |
| rs7248104 | 19 | TG | 0.0220 | 0.0034 | A | G | A | G | 0.0121710 | 0.0326019 | -0.0271575 | 0.0528830 | -0.0251083 | 0.0305887 |
| rs731839 | 19 | TG | 0.0220 | 0.0036 | A | G | A | G | 0.0113825 | 0.0340045 | 0.0316307 | 0.0551616 | -0.0923759 | 0.0325785 |
| rs6065906 | 20 | TG | 0.0530 | 0.0043 | T | C | T | C | 0.0590177 | 0.0404221 | 0.0277095 | 0.0664510 | 0.0481801 | 0.0381940 |
| rs5756931 | 22 | TG | 0.0200 | 0.0035 | C | T | C | T | -0.0589938 | 0.0326661 | 0.0018250 | 0.0535280 | -0.0457736 | 0.0307873 |
| Chr: chromosome; TC: total cholesterol; beta: regression coefficient; se: standard error; AS: aortic stenosis; AR: aortic regurgitation; MR: mitral regurgitation  Summary statistics related to SNP-TG extracted from Global Lipids Genetics Consortium 2013 [Discovery and refinement of loci associated with lipid levels; Nature Genetics volume 45, pages 1274–1283 (2013)] and corresponding SNP-outcomes estimations extracted from individual participants data of the UK Biobank. | | | | | | | | | | | | | | |
